# Beyond Passion and Perseverance: Review and Future Research Initiatives on the Science of Grit

**DOI:** 10.3389/fpsyg.2020.545526

**Published:** 2021-01-27

**Authors:** Jesus Alfonso D. Datu

**Affiliations:** Department of Special Education and Counselling, Integrated Centre for Well-Being, The Education University of Hong Kong, Hong Kong, China

**Keywords:** grit, passion, perseverance, performance outcomes, well-being

## Abstract

Grit, which is originally conceptualized as passion and perseverance for long-term goals, has been associated with optimal performance. Although previous meta-analytic and systematic reviews summarized how grit relates to performance outcomes, they possess considerable shortcomings, such as (a) absence of summary on the association of grit with well-being outcomes; (b) absence of discussion on social, psychological, and emotional mechanisms linking grit to well-being; and (c) lack of elaboration on how alternative models can resolve fundamental problems in the grit construct. This integrative review provides a comprehensive summary on the link of grit to performance and well-being outcomes. Importantly, it elaborates how alternative models can potentially address flaws in the existing grit theory. Future research directions are discussed on how to move forward the science of grit.

Psychological scientists have recognized the importance of exploring non-cognitive predictors of success in work, school, and other domains of life (Robbins et al., 2006; Duckworth and Yeager, 2015). One of the non-cognitive constructs gaining considerable attention in the existing literature is grit (Duckworth et al., 2007). Grit was originally defined as one's disposition to demonstrate perseverance and passion for long-term goals (Duckworth et al., 2007, p. 1087). Earlier studies have pointed out that grit was a higher-order construct composed of consistency of interests (ability to stick to a similar set of interests over time) and perseverance of effort (tendency to show diligence despite challenges or difficulties associated with pursuing a long-term goal), which could predict variety of positive performance outcomes including success in school, spelling quiz bee competition, and work among others (Duckworth et al., 2007; Duckworth and Quinn, 2009; Eskreis-Winkler et al., 2014).

There has been accumulating body of empirical evidences demonstrating the power of grit in predicting useful outcomes, success at school (Duckworth et al., 2007; Eskreis-Winkler et al., 2014; Strayhorn, 2014), positive organizational behaviors (Suzuki et al., 2015; Ceschi et al., 2016), and well-being (Vainio and Daukantaite, 2016; Li et al., 2018b; Datu et al., 2019). Moreover, Lee and Duckworth (2018) have accentuated the promising institutional benefits of “gritty” organizations.

Despite the growing interest on the role of grit in fostering success and well-being, a recent meta-analytic evidence has casted serious doubts regarding the theoretical validity of the originally theorized two-factor model of grit (Credé et al., 2017). The result of the authors' meta-analysis has shown that compared to consistency of interests, perseverance of effort is a stronger predictor of performance outcomes. Furthermore, the correlation of grit to academic achievement was not comparable to other known predictors of academic success. Studies have demonstrated that whereas perseverance was associated with various indicators of positive student functioning (i.e., academic adjustment and engagement), consistency was not significantly correlated with such outcomes (Bowman et al., 2015; Datu et al., 2016). The authors have also pointed out that the “primary utility of the grit construct may lie in the perseverance facet” (p. 113). Moreover, another review (Datu et al., 2017b) has pointed out possible cultural biases in the original grit framework. Indeed, more effort is needed to resolve empirical threats to the science of grit.

Although existing reviews on grit (Credé et al., 2017; Datu et al., 2017b; Credé, 2018; Lam and Zhou, 2019; Fernández et al., 2020) have provided comprehensive, detailed, and nuanced review on how grit tracks performance and other positive psychological outcomes, they have a number of considerable shortcomings. As these meta-analytic (Credé et al., 2017) and systematic (Datu et al., 2017b; Lam and Zhou, 2019; Fernández et al., 2020) reviews primarily focused on summarizing the association between grit and domain-specific performance (e.g., achievement in school contexts), conclusions have myopic implications for understanding the role of grit in optimizing other equally important outcomes, such as psychological well-being and physical health. Moreover, previous reviews have paid little attention to summarizing studies on psychological processes underpinning the complex link of grit to a wide range of outcomes. Most importantly, whereas these reviews have pinpointed fundamental theoretical flaws in the theorizing of grit, they failed to discuss alternative models that can potentially address such conceptual and measurement issues.

Therefore, this integrative review aims to provide a comprehensive summary regarding the measurement, correlates, and alternative models of the grit construct through answering these overarching questions: (1) What's wrong with the existing grit theory? (2) What are alternative theoretical models on grit? and (3) how can these alternative grit frameworks address fundamental flaws in the original grit theory? This article addresses such questions via (a) defining the grit construct; (b) summarizing literature on how grit is linked to adaptive performance, psychological, and physical outcomes as well as neural bases of grit; (c) providing a summary about the predictors of grit and its dimensions; (d) discussing theoretical flaws of the extant grit theory; (e) describing the caveats of continuously incorporating consistency of interests as a dimension of grit; (f) elaborating alternative conceptualization of grit, such as triarchic model of grit (Datu et al., 2017a, 2018a) and refined conceptualization of passion (Jachimowicz et al., 2018); and (g) elucidating how theoretical refinement of grit can potentially address its major conceptual shortcomings. Future research agenda and initiatives are elaborated.

## What is Grit?

Grit was operationalized as trait-level passion and perseverance for long-term aspirations (Duckworth et al., 2007). Specifically, Duckworth et al. (2007) conceptualized grit as a hierarchical construct underpinned by two interrelated dimensions, namely, consistency of interests and perseverance of effort. Consistency of interests entails constantly showing interest and efforts, whereas perseverance of effort involves demonstrating heightened intensity of persistence even after experiencing concrete setbacks or failures. The authors argued that grit was conceptually distinct from theoretically relevant constructs, such as conscientiousness and resilience. This multiphasic research has revealed that grit was associated with higher levels of educational attainment among adult participants in an online website, academic performance in undergraduate students, fulfillment of military training in a sample of freshmen cadets who enrolled in the US Military Academy, and advancement to more challenging stages of a national spelling quiz bee contest. Furthermore, grit accounts for ~1.4–6.3% of the variances in successful outcomes.

For the past several years, studies continued to adopt a trait-level conceptualization of grit emphasizing the relative stability of this construct regardless of situational conditions (Duckworth and Eskreis-Winkler, 2015). Even the existing measures on grit, such as the 12-item Original Grit Scale (Duckworth et al., 2007) and 8-item Short Grit Scale (Duckworth and Quinn, 2009) focused on assessing grit as an individual difference construct. Yet, although some studies have provided evidence about the incremental validity of grit over and beyond the effects of known predictors (i.e., conscientiousness and self-control) of achievement-related outcomes (Duckworth et al., 2007; Li et al., 2018c), there have been serious concerns on the psychometric validity of such grit scales (see Credé et al., 2017 and Datu et al., 2017b for reviews); more research is needed to understand the complex nature of grit.

Grit is also different from relevant psychological constructs (i.e., conscientiousness, need for achievement, and self-control). For instance, whereas conscientiousness is a Big Five personality trait characterized by diligence, achievement–orientation, and diligence (Soto et al., 2016), grit involves consistently working on a specific interest or endeavor and persisting over difficult tasks over a long period of time. In addition, while need for achievement (McClelland, 1961) involves strenuously spending effort and time on potentially “rewarding” activities, grit may not necessarily require incentives and feedback to boost desire for accomplishing long-term goals. Existing literature has also pointed out that grit was essentially distinct from self-control because the former appears to be more applicable to highly demanding contexts or situations, whereas the latter is more fitting for commonly faced day-to-day challenges (Duckworth and Gross, 2014; Eskreis-Winkler et al., 2016).

## Performance, Psychological, and Physical Benefits of Grit

There has been a steady inflation in the number of studies documenting the advantageous role of grit in facilitating success and well-being outcomes. This section summarizes previous research on how grit and its dimensions relate to various indicators of optimal performance, psychological well-being, and physical health.

### Grit and Academic Outcomes

There has been a considerable body of literature showing how grit may relate to school-related performance and behaviors. Gritty students are more likely to have higher levels of general academic achievement among university students in the United States (Duckworth et al., 2007; Duckworth and Quinn, 2009; Akos and Kretchmar, 2017); high school students in mainland China (Li et al., 2018c), secondary education students in the United States (Cosgrove et al., 2018; Park et al., 2018), Germany (Schmidt et al., 2019), Austria (Dumfart and Neubauer, 2016), and Russia (Tovar-García, 2017); course-specific academic achievement among military cadet samples in the United States (Mayer and Skimmyhorn, 2017); literacy achievement among primary school students (O'Neal et al., 2018); academic achievement in science in secondary school students in Australia (Hagger and Hamilton, 2019); performance in a national spelling bee contest (Duckworth et al., 2010); retention in selected undergraduate students in the United States (Saunders-Scott et al., 2018); academic engagement in selected university and high school students in the Philippines (Datu et al., 2016, 2018b); academic self-efficacy among university students in the Philippines (Datu et al., 2017a) and the United States (Renshaw and Bolognino, 2016); generalized self-efficacy (Renshaw and Bolognino, 2016); intellectual self-concept among selected twin sample in the United States (Tucker-Drob et al., 2016); emotional engagement among dual language learners in the United States (O'Neal et al., 2018); school-related motivation among Filipino, American, and Mexican American students (Eskreis-Winkler et al., 2014; Yeager et al., 2014; Piña-Watson et al., 2015; Datu et al., 2018b); learning engagement in selected mainland Chinese adolescents (Lan and Moscardino, 2019); test motivation among twins in the United States (Tucker-Drob et al., 2016); deliberate practice in optional and required practice in specific sports domains among selected athletes mostly from the North American context (Tedesqui and Young, 2017); satisfaction with e-learning systems among university students in Portugal (Aparicio et al., 2017); college satisfaction (Bowman et al., 2015); leadership skills among military cadets (Mayer and Skimmyhorn, 2017); mastery orientation (Tucker-Drob et al., 2016); meaningfulness of academic activities (Yeager et al., 2014); and growth mindset (Tucker-Drob et al., 2016). Furthermore, domain-specific grit in the school context is linked to elevated levels of academic performance among high school students in Germany (Schmidt et al., 2019) and middle school students in the United States (Clark and Malecki, 2019).

### Grit and Career Outcomes

Grit is associated with elevated levels of retention and teaching effectiveness among selected teachers in the United States (Robertson-Kraft and Duckworth, 2014) and career exploration self-efficacy in Filipino university students (Datu et al., 2017a), as well as fewer changes in career (Duckworth et al., 2007). Both perseverance and consistency are linked to higher work performance incentives in selected Chinese insurance employees (Zhong et al., 2018). In the context of selected cadets in the Corps, both perseverance and consistency were not associated with decision to become officers in the US Military institute (Jordan et al., 2015). In the case of surgical residents in the United States, grit has been found to be a key risk factor for attrition in the residency program (Burkhart et al., 2014). Also, residents whose scores fell below the median value are likely to report dissatisfaction with their residency programs.

### Grit and Work-Related Functioning

Gritty adults are more likely to demonstrate higher venture or business performance among entrepreneurs in the United States (Mueller et al., 2017), work engagement in Japanese adults (Suzuki et al., 2015), and positive leadership behaviors (Caza and Posner, 2019), as well as sports-related engagement in a sample of wheelchair basketball athletes in the United States (Martin et al., 2015) and selected male soccer athletes in Australia (Larkin et al., 2015). Conversely, grittier employees are less likely to experience work burnout and engage in counterproductive work behaviors (Ceschi et al., 2016). In a sample of surgical trainees in England, grit is related to lower levels of work burnout (Walker et al., 2016).

### Grit, Well-Being, and Positive Psychological Outcomes

Researchers have offered evidence on the well-being benefits of grittiness in diverse contexts. Grit is linked to higher levels of life satisfaction among undergraduate students in the United States (Renshaw and Bolognino, 2016); adults in Switzerland (Samson et al., 2011); selected male adults in the United States (Hammer and Good, 2010); employees in mainland China (Li et al., 2018a); university and post-graduate students in Sweden (Vainio and Daukantaite, 2016), as well as adults in South Korea (Jin and Kim, 2017); meaning in life among university students in the United States (Kleiman et al., 2013); psychological well-being among selected surgical residents in the United States (Salles et al., 2014) and student populations in Sweden (Vainio and Daukantaite, 2016); adolescent well-being in Australian and American youth samples (Kern et al., 2016); self-esteem (Hammer and Good, 2010) and optimism and mental health in selected US military recruits (Lovering et al., 2015); well-being in a sample of undergraduate and post-graduate students in England (Kannangara et al., 2018); money-conserving actions among US university students (Maddi et al., 2013); gratitude in selected Swiss adults (Samson et al., 2011) and Filipino high school students (Valdez and Datu, 2020); sense of coherence (Vainio and Daukantaite, 2016); subjective happiness (Samson et al., 2011); action-oriented tendencies of Romanian adult sample (Constantin et al., 2011); and harmony in life (Vainio and Daukantaite, 2016). Also, grit positively predicts prosocial behaviors in older adults in the United States (Wenner and Randall, 2016), as well as good habits in selected US university students (Feldman and Freitas, 2016). Domain-specific grit (i.e., academic grit) is linked to life satisfaction and school satisfaction among middle school students in the United States (Clark and Malecki, 2019). Moreover, analysis of data involving Western (e.g., United States, England, Russia, and Canada) and non-Western societies (e.g., Mexico, Malaysia, and China) shows that grit is positively correlated with both hedonic and eudemonic well-being (Disabato et al., 2016).

Individuals' passion and perseverance for long-term goals are also associated with various types of orientations to happiness (i.e., orientation toward pleasure, meaning, and engagement). Whereas, grit is positively correlated with orientations toward engagement and meaning among adults in the United States (Von Culin et al., 2014) and Japan (Suzuki et al., 2015), this construct is linked to lower levels of orientations to pleasure. These results indicate that grit may be linked to different types of orientations to happiness in Western and non-Western contexts.

Moreover, grit is associated with lower depression among undergraduate students in the United States (Anestis and Selby, 2015), adults in South Korea (Jin and Kim, 2017), military recruits in the United States (Lovering et al., 2015), and undergraduate students in Thailand (Musumari et al., 2018), as well as selected high school students in the Philippines (Datu et al., 2019); decreased levels of perceived stress in selected university and post-graduate students in England (Kannangara et al., 2018) and United States (Saunders-Scott et al., 2018); reduced chances of using alcohol and marijuana among Latino adolescents (Guerrero et al., 2016); decreased levels of anxiety (Musumari et al., 2018); lessened fear of being laughed at or gelatophobia (Samson et al., 2011); reduced anxiety sensitivity among young adults in the United States (Moshier et al., 2016); and lower suicidal ideation among US undergraduate students (Kleiman et al., 2013; White et al., 2017). Students with higher grit are also less likely to engage in problematic use of internet, as well as compulsive buying and gambling behaviors (Maddi et al., 2013).

### Neural Correlates of Grit

Prior studies have generated findings that have implications for understanding the neurobiological bases of grit. Drawing from functional magnetic resonance imaging approaches, one of the important regions in the brain that has been linked to grit is the medial prefrontal cortex (Myers et al., 2016; Wang et al., 2017). Specifically, neural connections in the medial prefrontal and rostral anterior cingulate cortices were associated with increased perseverance (Myers et al., 2016). There was also a negative correlation between grit and regional fractional amplitude of low-frequency fluctuations, which has been implicated for setting goals, implementing plans, self-control, and capacity to adaptively interpret setbacks. Using a voxel-based morphometric design, Wang et al. (2018) have shown that regional gray matter volume in the right putamen is linked to increased levels of grit. In addition, an event-related potential study has demonstrated that undergraduate students with higher scores on perseverance of effort subscale tend to have lower reaction times in the Attention Network Task and lower mean difference in N1 amplitudes (Kalia et al., 2018), which suggest positive correlations between grit and task-related attention. Vlasova et al. (2018) have also revealed that “structural integrity in white matter pathways,” which was commonly implicated for emotion regulation and resilience, was positively correlated with grit among depressed adults.

### Moderation Studies on Grit on Maladaptive Functioning

Another promising line of evidence on grit alludes to the buffering role of grit. For instance, a synergistic interplay between grit and gratitude diminishes suicidal ideation through boosting meaning in life in selected US undergraduate students (Kleiman et al., 2013). Also, grit moderates the association of negative life events on suicidal ideation such that for students who scored lower in grit, negative events can promote suicidal ideation among undergraduate students in the United States (Blalock et al., 2015). Yet, little is known on whether long-term exposure to undesirable environmental conditions or events can dampen the protective role of grit on well-being outcomes. In addition, rumination heightened suicidal ideation for undergraduate students who had lower scores on grit (White et al., 2017).

Grit also serves as a protective factor against the psychological hazards (i.e., suicidal ideation) of hopelessness in selected US military personnel (Pennings et al., 2015). Furthermore, in situations where students do not have positive relationships with teachers, demonstrating grit is linked to elevated levels of school engagement and satisfaction with school (Lan and Moscardino, 2019). Lastly, for individuals with average and low scores on expressive suppression (an emotion regulation strategy that involves intentionally hiding or reducing one's emotional states; Gross and Levenson, 1997), showing elevated levels of consistency of interests may not result in maladaptive eating attitude and actions (Knauft et al., 2019).

### Grit and Physical Health

Contemporary investigations have also explored the link of grit to various indices of optimal physical health. Research suggests that grittier individuals are more likely to have a habitual exercise routine (Reed et al., 2013) and decreased chances of experiencing food insecurity (Nikolaus et al., 2019). In the case of adolescents and young adults who are facing long-term medical illnesses, grit is linked to lower levels of depression and anxiety, as well as emotional well-being (Sharkey et al., 2017). Composite grit scores are also negatively correlated with deficits in executive functioning, cognitive failures, and symptom severity among selected diagnosed undergraduate students with attention-deficit/hyperactivity disorder (ADHD) in Canada (Gray et al., 2015). Similarly, demonstrating increased perseverance of effort is associated with optimal neurocognitive functioning in a sample of people living with human immunodeficiency virus in the United States (Moore et al., 2018).

Although results from previous studies generally suggest that overall grit can facilitate success and well-being in various domains of functioning, it may be challenging to delineate what aspects of grittiness may promote desirable outcomes. In the succeeding section, I summarized studies demonstrating how each dimension of grit differentially relates to various domains of optimal performance, psychological health, and physical well-being.

## Grit Dimensions as Differential Predictors of Optimal Functioning

Previous research has offered robust evidence on the importance of perseverance of effort in fostering positive performance, psychological, and well-being outcomes. Perseverance is associated with higher levels of academic achievement among undergraduate students in the United States (Chang, 2014; Wolters and Hussain, 2015; Muenks et al., 2017), adolescent twins in the United Kingdom (Rimfeld et al., 2016), secondary school students in Germany (Steinmayr et al., 2018), adolescent students in Finland (Tang et al., 2019), associate degree students in Hong Kong (Lee, 2017), and selected high school students (Li et al., 2018c) and primary school students (Jiang et al., 2019) in mainland China; subjective academic performance in selected undergraduate students in Australia (Hodge et al., 2018); academic adjustment in the United States (Bowman et al., 2015); academic self-efficacy in US university students (Wolters and Hussain, 2015) and Filipino undergraduate students (Datu et al., 2017a); all achievement goal orientations (i.e., mastery–approach, mastery–avoidance, performance–approach, and performance–avoidance goals) among selected university students in the United States and mainland China (Chen et al., 2018); generalized self-efficacy in a sample of selected cadets in the United States (Jordan et al., 2015); entrepreneurial success in selected entrepreneurs in Australia (Mooradian et al., 2016); and academic self-regulation strategies, such as cognitive, metacognitive, motivational, and time and environment strategies among undergraduate students in the United States (Wolters and Hussain, 2015). This dimension of grit is also linked to higher levels of job satisfaction in a sample of employed students in the United States (Meriac et al., 2015), orientations to engagement among Japanese adults (Suzuki et al., 2015), general self-esteem among university students in the United States (Weisskirch, 2018), and mindfulness in selected Thai and New Zealand university students (Raphiphatthana et al., 2019). In a sample of adults in the United States, compared to consistency of interests, which is associated with weaker indications of sympathetic activity, perseverance of effort is related to increased activation of autonomic nervous system (Silvia et al., 2013).

Consistent with the arguments on the performance and well-being benefits of perseverance, studies show that this facet of grit is negatively correlated with turnover intentions (Meriac et al., 2015), academic maladjustment (Hwang et al., 2018), perceived stress (Meriac et al., 2015; Lee, 2017; Mullen and Crowe, 2018; Zhong et al., 2018), burnout (Mullen and Crowe, 2018; Zhong et al., 2018), and academic procrastination among selected undergraduate students in Italy (Pierro et al., 2011). School-specific perseverance is also positively correlated with academic achievement (Schmidt et al., 2019).

On the other hand, research indicates that consistency of interests is not related to academic performance (Chang, 2014; Wolters and Hussain, 2015; Rimfeld et al., 2016; Lee, 2017; Hodge et al., 2018; Jiang et al., 2019; Tang et al., 2019), academic maladjustment (Hwang et al., 2018), most academic self-regulation approaches except time and environment management strategies (Wolters and Hussain, 2015), job satisfaction (Meriac et al., 2015), and turnover intent (Meriac et al., 2015). Few researches provide insights regarding the benefits of consistency by showing how this facet of grit may be linked to higher academic performance (Li et al., 2018c), elevated self-esteem and adaptive learning strategies (Weisskirch, 2018), lower likelihood of shifting to another major or vocational tracks (Bowman et al., 2015), reduced performance–avoidance goals (Chen et al., 2018), decreased bulimia and body satisfaction among selected adults from eating disorder treatment facilities (Knauft et al., 2019), reduced perceived stress (Meriac et al., 2015; Lee, 2017; Mullen and Crowe, 2018), decreased levels of academic procrastination (Pierro et al., 2011), and lower levels of burnout (Mullen and Crowe, 2018; Zhong et al., 2018).

## Why Does Grit Predict Positive Outcomes?

**Figure 1:**
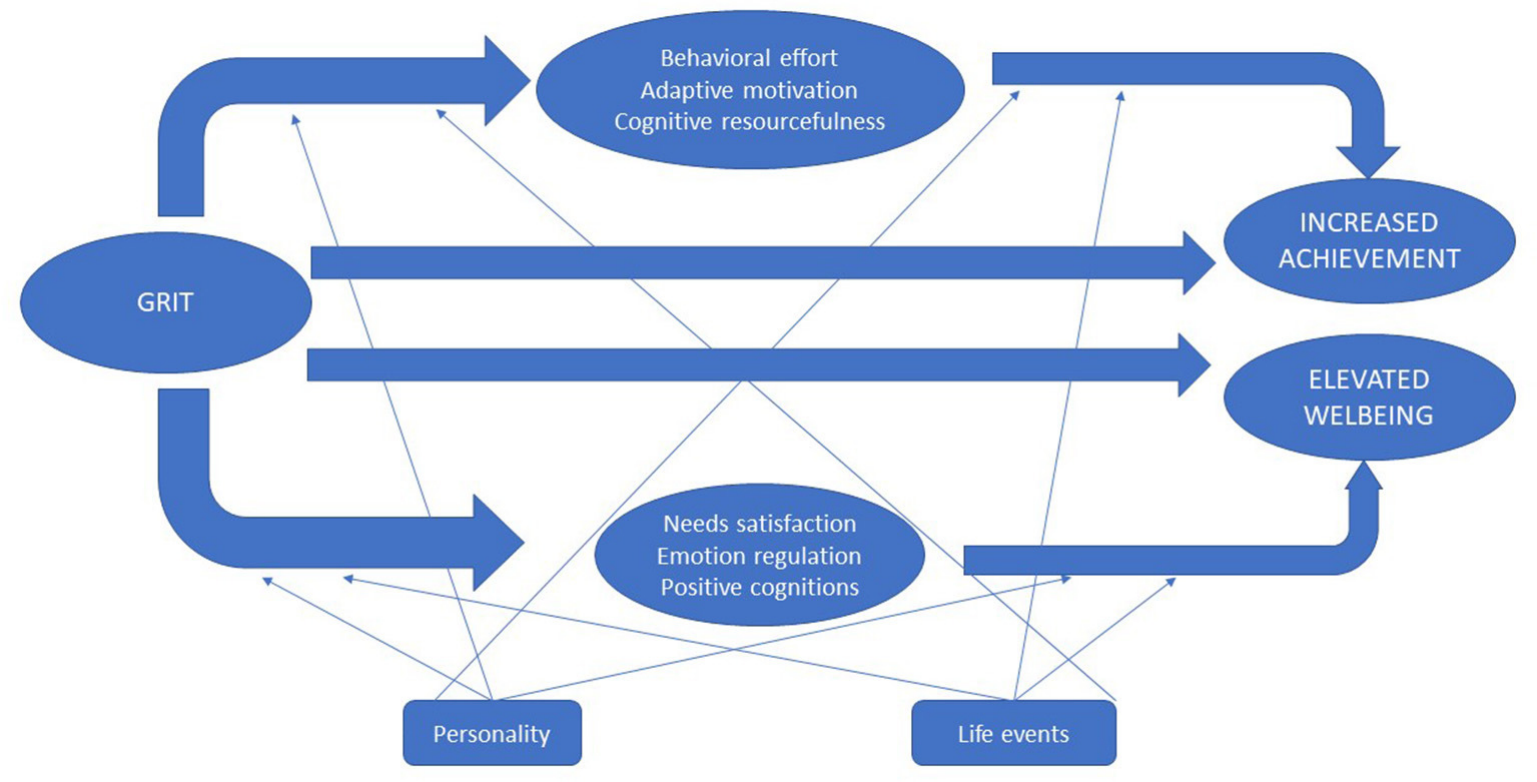
The optimal performance and health (OPAH) model of grit.

As grit and its dimensions have linked to increased achievement in various domains of performance and well-being, past studies have identified precise mechanisms underscoring the positive impacts of grit on desirable outcomes. Based on the results from previous investigations, this review proposes the optimal performance and health (OPAH) model of grit (Figure 1), which summarizes processes involved in the anticipated benefits of grittiness, as well as intrinsic (i.e., personality) and extrinsic (e.g., life events) factors that moderate the link of grit to positive outcomes.

The first path of the diagram demonstrates why grit can optimize elevated levels of achievement in specific domains of performance. It is likely that persevering for long-term goals can predict achievement because existing studies have shown actual effort invested in specific activities mediated the link of grit to positive academic outcomes (Duckworth et al., 2010; Hagger and Hamilton, 2019). Grittier students, for instance, advanced to more complicated rounds in a national spelling bee because they have spent more effort and time in keenly preparing for the said competition (Duckworth et al., 2010). In the same way, the capacity of perseverance to boost effort in accomplishing science-related tasks explains why this facet of grit predicts increased achievement in Science (Hagger and Hamilton, 2019). Moreover, perseverance is related to increased time and environment management academic regulation strategies (Wolters and Hussain, 2015) and active participation in school-related tasks (Datu et al., 2016). Thus, “behavioral effort” is operationalized as one the psychological processes underpinning the effects of grit on achievement outcomes.

Moreover, results from past studies point to the role that “adaptive motivation” plays in grit–success relationship. This type of process variable encompasses one's drive or desire to achieve optimal levels of performance. Corroborating this perspective, grit is positively linked to mastery–approach goals, mastery–avoidance goals, performance–approach goals, and performance–avoidance in selected undergraduate students in the United States and mainland China (Chen et al., 2018), as well as autonomous motivation among Filipino high school students (Datu et al., 2018b). Research also demonstrates that autonomous motivation mediates the positive associations of grit with agentic, behavioral, cognitive, and emotional engagement (Datu et al., 2018b).

“Cognitive resourcefulness” characterizes another type of variable serving as a mechanism that explains why grit may be linked to appealing performance outcomes. It covers any construct that necessitates usage of cognitive resources to successfully accomplish a wide range of tasks in various settings. For instance, research shows that perseverance is linked to increased entrepreneurial success due to the mediating role of innovativeness (Mooradian et al., 2016). In addition, perseverance of effort is related to higher levels of cognitive and metacognitive study strategies (Wolters and Hussain, 2015) and elevated cognitive engagement (Datu et al., 2018a,b). Moreover, grit enhances subsequent desirable changes in goal attainment among samples from different societies (Sheldon et al., 2015).

The second path in the diagram illustrates how grit can foster different well-being outcomes through concrete psychological processes. One of the mechanisms underlying the complex link of grit to well-being involves “needs satisfaction.” This type of process variable refers to fulfillment of basic psychological needs (c.f., autonomy, relatedness, and competence; Ryan, 1995). It is reasonable to expect that satisfaction of needs can mediate the association of grit with well-being because self-determination theory has pointed out that satisfying psychological needs can serve as a route to achieve intrinsic motivation, well-being, and optimal psychological functioning (Ryan, 1995; Deci and Ryan, 2000; Ryan and Deci, 2000). Research dovetails with this prediction as satisfaction of basic needs for autonomy and competence mediate the positive influence of grit on well-being (Jin and Kim, 2017).

Another psychological process that can elucidate why grit may increase well-being outcomes is “emotion regulation.” This category of mediating variables pertains to one's ability to manage emotions in different situations, such as (a) cognitive reappraisal, which involves modifying the interpretation of a specific situation to change its emotional consequences; and (b) expressive suppression, which entails decreasing or hiding one's true emotional states (Gross and Levenson, 1997). Consistent with this premise, perseverance of effort and consistency of interests are linked to higher levels of cognitive reappraisal (Knauft et al., 2019; Valdez and Datu, 2020). Yet, consistency is related to lower levels of expressive suppression. Also, cognitive reappraisal mediates the association of grit with psychological flourishing (Valdez and Datu, 2020).

The third classification of process variables underpinning the linkage of grit to well-being outcomes is “positive cognitions.” This type of variable encompasses one's capability to espouse positive thoughts about self, others, and environment. Echoing this prediction, research demonstrates how constructs belonging to this category of mediating variable mediate the association of grit on well-being outcomes, such as meaning in life (Kleiman et al., 2013), mindfulness (Li et al., 2018b), and self-esteem (Li et al., 2018a). Moreover, authenticity (perceived feelings of being connected to one's self; Wood et al., 2008) and sense of coherence (degree to which one's self and the world are sensible, meaningful, and controllable; Antonovsky and Sagy, 1986) mediate the association of grit with subjective and psychological well-being outcomes (Vainio and Daukantaite, 2016). For adolescents and young adults with long-term medical conditions, grit is associated with well-being (i.e., higher emotional well-being and lower depression, as well as anxiety) because this construct is linked to reduced perceptions that illnesses can disrupt their daily activities (Sharkey et al., 2017).

Aside from pinpointing specific psychological mechanisms that can underscore the benefits of grit on well-being, existing studies indicate how dispositional and contextual variables may strengthen or attenuate the effects of grit on various outcomes. It is likely that personality traits can moderate the anticipated impacts of grit on psychological functioning. For instance, a synergistic interaction between gratitude and grit increases meaning in life, which in turn decreases suicidal ideation (Kleiman et al., 2013). Furthermore, life events can also interact with grit to yield differential effects on well-being outcomes. Supporting this prediction, research reveals that negative life events can lead to suicidal ideation for individuals with lower inclination to show passion and perseverance for long-term goals (Blalock et al., 2015).

In general, Figure 1 recapitulates concrete cognitive, emotional, motivational, and behavioral processes that elucidate how and why grit cultivates optimal levels of performance outcomes and psychological health. Although this diagram offers a brief overview of precise psychological mechanisms linking grit to meaningful outcomes, note that this model is generated from past investigations that relied on the original two-factor model of grit (Duckworth et al., 2007), which attracted numerous conceptual and methodological criticisms on the grit construct.

## Demographic, Psychological, and Social Factors Associated with Grit

Prior studies have demonstrated different demographic and psychological predictors of grit. In terms of demographic factors that relate to grit, there is evidence showing that age and length of work experience are associated with increased levels of grit over time (Duckworth et al., 2007; Camp et al., 2018). However, Usher et al. (2019) have shown that older students and those with low socioeconomic status tend to report low levels of grit. These findings indicate that the role of specific factors, such as age on grit is inconclusive.

Previous investigations have also shown specific psychological factors that may be linked to grit. For example, positive affect and purpose commitment have been associated with higher levels of grit in selected American and Canadian students (Hill et al., 2014). In a sample of Latina/o students, higher levels of hope positively predicted grit, whereas higher levels of search for meaning in life negatively predicted grit (Vela et al., 2015). Further, mindfulness positively predicted grit (Raphiphatthana et al., 2018, 2019; Vela et al., 2018). There is also evidence showing that whereas experiencing fitness-related pride attributed to one's own effort (i.e., authentic fitness-related pride) was found to predict grit, fitness-related success attributed to one's innate ability or superiority (i.e., hubristic fitness-related pride) negatively predicted persistence and interest toward goal attainment (Gilchrist et al., 2018). Belief in free will has been also associated with increased perseverance in selected Chinese adolescents (Li et al., 2018d).

Further, Armstrong et al. (2018) have demonstrated six self-regulation strategies, such as temporal perspective, perpetual evaluation, motivational orientation, strength and resource gathering, system thinking, and framing, were linked to higher levels of grit. In a national sample of American college students, Sriram et al. (2018) have shown that others-focused purpose, success-focused purpose, time spent in socializing, time spent in academic activities, and religious beliefs positively predict grit. Spirituality has also shown direct and positive effects on grit via the mediating role of employment hope among underemployed urban African Americans (Hodge et al., 2019).

There are studies showcasing the link of social factors to grit in different contexts. Whereas, excessively controlling parenting behaviors negatively predicted grit, parental involvement positively predicted grit (Howard et al., 2019). Grit also mediates the link of parenting-related behaviors to academic success (Howard et al., 2019). Relatedness to different social agents (i.e., parents, peers, and teachers) has different pattern of associations with grit and its dimensions with relatedness to teachers positively predicting perseverance, consistency, and overall grit, while relatedness to parents positively predicting consistency and overall grit (Datu, 2017).

## What's Wrong with the Existing Grit Theory?

The considerable fund of empirical evidences showcasing the performance, well-being, and psychological advantages of fostering grit does not come without substantial controversies. This section summarizes results from previous studies highlighting major flaws in the extant theorizing and measurement of grit. Specifically, this is divided into four subsections, namely, measurement issues, theoretical validity, cultural bias in grit, and consistency or adaptability.

### Measurement Issues

The existing grit framework (Duckworth et al., 2007) is continuously plagued by cumulative number of investigations indicating major problems in measuring the grit construct. Studies reveal psychometric issues on the items belonging to the consistency of interests dimension of grit in non-Western societies, such as Japan (Yoshitsu and Nishikawa, 2013; Datu et al., 2020) and the Philippines (Datu et al., 2016, 2017a, 2018a). Specific items (“My interests change from year to year” and “I have difficulty maintaining my focus on projects that take more than a few months to complete”) on consistency, for instance, were omitted in the investigation of Suzuki et al. (2015) because these items did not load onto the consistency dimension of grit in a preceding study (Yoshitsu and Nishikawa, 2013). Similarly, in an investigation conducted among selected Romanian adults, correlating two error terms (indicating issues in the wording of items) resulted in an acceptable measurement model of grit (Ion et al., 2017). In a sample of school-based counselors in the United States by Mullen and Crowe (2018), one item in the perseverance of effort dimension (“Setbacks don't discourage me.”) had a poor factor loading (0.15). Including this item in examining the internal consistency of perseverance of effort facet of grit resulted in a low Cronbach α coefficient (α = 0.57). Moreover, the two-factor model of grit generated from the 12-item original grit scale was not confirmed in a sample of athletes in North America (Tedesqui and Young, 2017). There is also evidence showing that the consistency subscale had poor reliability coefficients in non-Western and collectivist settings (Disabato et al., 2019).

When the hierarchical model of grit underpinned by perseverance and consistency as first-order latent factors was tested in selected Filipino university and high school student samples (Datu et al., 2016), results have shown that the scores from the said measurement model did not achieve an adequate fit. The factor structure of original grit model also differs among secondary school and undergraduate student samples (Muenks et al., 2017). Analyses of grit scales (e.g., Short Grit and Original Grit Scales) using item response theory (IRT) approaches also showed mixed evidence regarding the dimensionality of grit with one research demonstrating its unidimensionality (Areepattamannil and Khine, 2018), while other studies demonstrating its multidimensionality (Tyumeneva et al., 2019; Gonzalez et al., 2020). Furthermore, existing measure of grit is plagued by social desirability bias (DiMenichi and Richmond, 2015). Much like what other studies have consistently noted (Datu et al., 2016, 2018a; Datu and McInerney, 2017; Muenks et al., 2017), there is a need to improve existing measures of grit.

Adopting an extension procedures of confirmatory factor analysis (CFA) that enabled a more nuanced account on how dimensions of grit relate to conscientiousness while taking into consideration the hierarchical and lower-level factors of both constructs across German student and adult samples, Schmidt et al. (2018) have shown that perseverance exhibited strong association with common factors (95% shared variance) indicating this dimension of grit could be subsumed under the proactive aspect of conscientiousness (Roberts et al., 2005). Perseverance was also strongly linked to the industriousness facet of conscientiousness. Furthermore, consistency demonstrated a high relationship to the common factors of conscientiousness but more specifically related to the self-discipline facet of conscientiousness. Another research also demonstrated that the grit construct overlapped with self-control (Gonzalez et al., 2020). These findings further offer additional evidence on the conceptual resemblance of grit with conscientiousness and self-control.

Contrary to the fundamental tenets of original grit theory, previous research alludes to the possibility that consistency of interests does not belong to the higher-order grit latent construct (Abuhassàn and Bates, 2015). There is a significant but weak correlation between perseverance and consistency (Constantin et al., 2011; Jordan et al., 2015; Meriac et al., 2015; Wolters and Hussain, 2015; Tedesqui and Young, 2017). Indeed, measurement issues raised in the aforementioned studies reinforces the theoretical value of perseverance but not consistency in understanding the nature of grit.

Taken together, prior studies suggest considerable issues not only with its specific items but also with the entire consistency subscale of existing grit measures (i.e., Grit-O and Grit-S). There are different reasons that might account for psychometric problems on the consistency subscale. For example, whereas the consistency of interests has been conceptualized as one's tendency to constantly stick to interests that enable fulfillment of long-term ambitions (Duckworth et al., 2007), items in the extant grit scales tap into one's capacity to maintain focus on ideas or projects, which may not necessarily capture activities or endeavors that individuals consider meaningful. As previous investigations (Carlson et al., 2011; Zhang et al., 2016; Lee and Duckworth, 2018) have shown that scales with negatively worded or reverse-scored items are likely to yield undesirable impact on the overall psychometric validity of such measures, it is possible that the nature of wording in the consistency subscale in existing grit measures contributes to the fundamental problems in assessing grit.

### Theoretical Validity

The existing two-factor model of grit has a few considerable flaws. One of the major shortcomings of theory includes the inability of consistency of interests to predict success and other meaningful outcomes. Dovetailing with this argument, previous investigations have found that consistency of interests does not relate to optimal functioning, such as academic achievement (Akos and Kretchmar, 2017; Muenks et al., 2017; Jiang et al., 2019), academic engagement (Datu et al., 2016, 2018a), and generalized self-efficacy (Jordan et al., 2015). Consistency is negatively correlated with an adaptive orientation to happiness (i.e., orientation to engagement; Suzuki et al., 2015). Overall grit score was not significantly correlated with achievement scores (Chang, 2014) and life satisfaction (Martin et al., 2015).

Moreover, although grit is positively correlated with in-role performance and organizational citizenship behaviors, it has limited predictive validity relative to Big Five personality factors on work-related (i.e., in-role performance, organizational citizenship behaviors, and counterproductive work behaviors) and well-being outcomes among selected Romanian adults (Ion et al., 2017). Although perseverance positively predicts academic performance, academic engagement and self-regulation were more strongly linked to achievement outcomes (Muenks et al., 2017). Conscientiousness also emerged as the strongest correlate of achievement when grit, intelligence, Big Five personality factors, self-efficacy, motivation, and test anxiety were entered as predictors of such outcome (Dumfart and Neubauer, 2016). Compared with grit, conscientiousness served as a stronger predictor of military cadets' academic and non-academic outcomes (Mayer and Skimmyhorn, 2017). After controlling the effects of extraversion, neuroticism, agreeableness, and openness, composite grit score did not predict academic achievement, academic recognition, honors, and rule-violating actions (Ivcevic and Brackett, 2014). Compared to grit, hardiness has been found to be a robust negative predictor of maladaptive internet usage and compulsive behaviors (Maddi et al., 2013). Similarly, only the perseverance dimension of grit predicted academic performance after controlling for the influence of conscientiousness (Rimfeld et al., 2016; Credé et al., 2017). Further, a recent study (Usher et al., 2019) demonstrated that grit did not predict academic achievement when relevant demographic covariates, such as gender, year level, and socioeconomic status were added as covariates among elementary and middle school students in the United States.

Vazsonyi et al. (2019) have offered evidence on the (a) unidimensionality of grit construct with latent method factors to characterize positively and negatively worded items and (b) substantial overlap between grit and self-control. Moreover, contrary to the theoretical predictions of Duckworth and Gross (2014) about the differential roles of both constructs on desirable performance outcomes (e.g., possessing high grit and low self-control will be strongly linked to approach outcomes, whereas having high self-control and low self-control will strongly relate to avoidance outcomes), there was no difference on how grit and self-control relate to various outcomes, such as approach or avoidance temperament, present goals, and past goals. Yet, as the study used cross-sectional research design and self-reported measures in exploring the internal, discriminant, and construct validity of existing grit scales, results may provide myopic evidence on the measurement problems associated with grit along with its conceptual similarity with self-control.

### Cultural Bias in Grit

Aside from budding line of empirical evidences magnifying the theoretical shortcomings of the original grit framework, another critical problem in the existing grit literature is the excessive quantity of published articles that examined the nature, antecedents, and consequences of grit in countries regarded by previous researchers (Henrich et al., 2010) as WEIRD (Western, educated, industrialized, rich, and democratic) societies. Given that citizens in non-Western cultures comprises more than 60% of the world's population, it is imprecise and deceptive to automatically generalize that findings from previous research might apply to individuals from non-WEIRD contexts. Despite the relative significance of advancing grit literature in non-Western cultures, there is still marked paucity of studies investigating the role of grit's facets in collectivist countries.

To date, studies on grit have focused on exploring the role that grit plays in optimizing optimal academic, work-related, and well-being outcomes in some non-Western societies, such as Japan (Suzuki et al., 2015), China (Li et al., 2018a,b,c), Hong Kong (Lee, 2017; Datu and Fong, 2018), mainland China (Chen et al., 2018), Philippines (Datu et al., 2016, 2017a, 2018a,b, 2019, 2020), South Korea (Jin and Kim, 2017), and Thailand (Raphiphatthana et al., 2019), among others. Without additional investigations on the antecedents and consequences of grit in non-WEIRD settings, it may be challenging to offer convincing insights on how grit dimensions may differentially relate to performance and positive psychological outcomes in various societies. Moreover, few studies (Chen et al., 2018), thus far, have adopted a cross-cultural design in exploring how grit relates to academic and well-being outcomes (Chen et al., 2018; Disabato et al., 2019; Raphiphatthana et al., 2019; Datu et al., 2020).

### Consistency or Adaptability

As serious issues are raised on the heuristic value of consistency of interests in the validity of grit construct (e.g., Credé et al., 2017; Schmidt et al., 2018; Datu et al., 2020), it is important to discuss how and why consistency may not necessarily contribute to successful accomplishment of long-term goals. This subsection draws from diverse perspectives to elaborate the mechanisms underscoring the non-significant role of consistency in catalyzing achievement and well-being in various contexts.

First, as it is likely that individuals can face many demanding situations in the pursuit of long-term goals, shifting from one interest to another and even relaxing difficult goals can serve as equally beneficial approaches to achieve optimal performance (Kashdan and Rottenberg, 2010; Dreisbach and Fröber, 2019). In situations where failure is inevitable, showing passion and persistence may not eventually pay off. Instead of achieving visible indicators success, persevering in face of failure might even result in irreparable damages or losses. Blind persistence (Baumeister et al., 2003) therefore is not an ideal strategy when one is facing tasks that could not be realistically completed. Studies have underscored the practical importance of knowing when to jettison life goals that are bound to fail (Baumeister et al., 2003; Lucas et al., 2015). Other research has emphasized the equally appealing value of dropping originally identified long-term goals in lieu of more realistic aspirations (Wolters and Hussain, 2015; Datu and McInerney, 2017; Datu et al., 2018a).

Second, individuals who espouse higher interdependent self-construal are inclined to exhibit self-variability, a tendency to engage in behaviors based on situational demands or cues (Suh, 2007; Vignoles et al., 2016). Salience of self-variability can potentially account for why consistency of interests is not linked to well-being in many collectivist societies (Disabato et al., 2019). Instead, adaptability is linked to higher levels of self-efficacy, academic engagement, motivation, and well-being in Filipino high school students (Datu et al., 2017a,b). These findings underscore the importance of calibrating interests, behaviors, and goals contingent on one's context or situation.

Third, from the vantage point of psychosocial theory of development (Erikson, 1982), human beings go through distinct developmental stages requiring various sets of psychological and interpersonal competencies. If one consistently sticks to specific interests or goals that may not facilitate successful resolution of a psychosocial crisis (e.g., identity vs. role confusion), he or she may end up losing trail of succeeding more advance developmental stages, which is detrimental to psychological health. For example, an adolescent who is constantly showing excessive interests in playing online computer games but aspiring to become a medical doctor may face difficulties in achieving better academic performance. This, in turn, can cause a serious toll on his vocational identity (thus leading to inability to achieve a sense of identity) as it may be challenging to be admitted in good university degree programs with mediocre performance. Consistent with this perspective, research indicates that in the case of older adults, it is likely that grit may yield desirable effects on prosocial behaviors because espousing this trait expands their opportunity to achieve their developmental tasks (Wenner and Randall, 2016). To the extent that grit afford adults with concrete prospects of being productive members of communities, this trait can optimize successful resolution of psychosocial developmental stage, such as generativity vs. stagnation (Erikson, 1982).

Fourth, whereas the original grit theory (Duckworth et al., 2007) highlighted the role of consistency, existing studies indirectly point to the benefits of adaptability (e.g., calibrating one's interests or goals based on situational demands) in achieving long-term aspirations. Dovetailing with this prediction, research suggests that grit may have state-like features (DiMenichi and Richmond, 2015) and is more strongly associated with the openness to experience compared to conscientiousness in Japanese adults (Suzuki et al., 2015). Furthermore, a study has shown that compared to other grit profiles (i.e., high perseverance and high consistency as well as low perseverance and high consistency), students belonging to a profile characterized by high perseverance and low consistency had significantly higher scores on hope and lower scores on anxiety and shame (Datu and Fong, 2018).

Indeed, the aforementioned theoretical premises indicate that consistency may have limited predictive power in shaping meaningful outcomes. Instead, it is reasonable to propose that adaptability can facilitate successful fulfillment of long-term goals. However, more research is needed to understand how replacing consistency with adaptability can improve the theoretical validity of grit construct.

## Moving Forward with the Science of Grit

The identified controversies on the theorizing and measurement of grit open considerable spaces for refining the existing grit framework. Evidences clearly suggest that improving the two-factor model of grit can advance our understanding on the nature, and antecedents, and consequences of grittiness in various contexts (Credé et al., 2017; Datu et al., 2017b).

### Designing Alternative Conceptualizations and Measures of Grit

One of the recent attempts to improve grit framework involves development of the triarchic model of grit (Datu et al., 2017a, 2018a), which conceptualizes grit as a disposition to show passion, perseverance, and adaptability for long-term goals. Like the original grit model (Duckworth et al., 2007), this model of grit has emphasized the importance of perseverance of effort and consistency of interests in achieving very challenging distal goals. However, the revised model highlights the theoretical value of incorporating adaptability to situations, defined as capacity to constantly calibrate one's interests and actions depending on situational and contextual factors. Triarchic Model of Grit Scale (TMGS; Datu et al., 2017a) was developed based on the items under the perseverance and consistency dimension of the Short Grit Scale (Duckworth and Quinn, 2009) and newly formulated items on adaptability. Research has provided preliminary evidence regarding the validity, reliability, and gender invariance of TMGS among Filipino undergraduate and high school student samples (Datu et al., 2017a, 2018b).

Triarchic model of grit dimensions has been found to be differentially linked to positive academic, psychological, and well-being outcomes. For instance, whereas perseverance and adaptability are associated with higher levels of self-efficacy in various domains of performance (i.e., academic, career exploration, and talent development self-efficacy), consistency was not (Datu et al., 2017a). Similarly, while adaptability and perseverance are linked to optimal school functioning, such as autonomous motivation, controlled motivation, and all components of academic engagement (i.e., agentic, behavioral, cognitive, and emotional engagement), consistency was linked only to behavioral engagement (Datu et al., 2018b). Although all dimensions of grit are positively correlated with life satisfaction, only perseverance and adaptability are associated with well-being outcomes (Datu et al., 2018b; Datu and Restubog, 2020). Datu and Restubog (2020) have shown that these TMG dimensions were linked to increased positive emotions due to the intermediate variable—social emotional learning. Both perseverance and adaptability are also related to increased well-being not just in the context of Filipino students but also in selected Japanese and Polish undergraduate students (Datu et al., 2020).

Although there is a reason to argue that triarchic model of grit (Datu et al., 2017a, 2018a) may potentially address theoretical and methodological issues raised in the existing two-factor model of grit (Duckworth et al., 2007), this program of research remains to be at the embryonic phase. For example, to what extent does this revised model of grit incrementally predict performance outcomes beyond and above the effects of relevant personality factors, such as openness to experience and conscientiousness? Can triarchic model of grit predict overall and domain-specific academic achievement beyond the influence of theoretically related constructs, such as academic self-regulation, academic self-efficacy, motivation, and engagement? Do consistency, perseverance, and adaptability serve as differential predictors of success and well-being? What psychological processes underpin the hypothesized educational benefits of this grit model? In what ways do different grit profiles relate to achievement and well-being outcomes?

Furthermore, a more recent approach involves polishing the consistency (a.k.a. passion) dimension of grit. Jachimowicz et al. (2018) refined the passion facet of grit through redefining this dimension as “as a strong feeling toward a personally important value/preference that motivates intentions and behaviors to express that value/preference” (p. 9980). The study demonstrates that combining perseverance and passion is linked to higher levels of work-related and academic performance. However, recent research has pointed out theoretical and measurement issues on the validity of the total score generated by their scale measuring perseverance of effort and passion, to assess perseverance (Credé, 2019).

Despite the potential benefits of “redefining” the consistency dimension of grit, future research is necessary to strengthen the evidence on the validity of this modified grit framework. For instance, how does the consistency facet of grit differ from the harmonious passion conceptualized in the dualistic model of passion (Vallerand et al., 2003)? Will this modified grit model incrementally predict achievement and optimal psychological outcomes beyond the contributions of different types of passion (e.g., obsessive and harmonious passion), Big Five personality factors, domain-specific motivation, and self-regulation? In what ways do the recently conceptualized passion facet and perseverance relate to various forms of well-being (i.e., subjective well-being, psychological well-being, psychological flourishing, and physical health)? Clearly, more studies are needed to understand the nature of grit given the growing body of evidence raising criticisms on the original two-factor model (Duckworth et al., 2007) and recently conceptualized models, such as the triarchic model of grit (Datu et al., 2017a, 2018a) and perseverance + passion model of grit (Jachimowicz et al., 2018).

Additional investigations are warranted to explore how domain-specific forms of grit may contribute to objective and subjective performance indicators. Past research has also underpinned the significance of assessing domain specificity of grit (Duckworth et al., 2007; Duckworth and Quinn, 2009; Wolters and Hussain, 2015; Clark and Malecki, 2019; Cormier et al., 2019). For instance, Clark and Malecki (2019) have shown that academic grit had incremental validity over domain-general grit scores in predicting academic performance and well-being outcomes (i.e., life and school satisfaction). Similarly, school-specific grit incrementally predicts academic achievement beyond the influence of domain-general grit and gender (Cormier et al., 2019). Other researchers have developed and validated domain-specific grit scales for foreign language learning (Ebadi et al., 2018). Yet, it is evident that except for the study of Ebadi et al. (2018), existing studies primarily focused on the consequences of domain-specific grit in Western societies (e.g., United States). Addressing this methodological grit entails exploring the role of domain-specific grit in non-Western and collectivist contexts to provide evidence about the cross-cultural applicability of grit in various cultural settings.

On top of refining extant grit scales, future studies can adopt alternative approaches in assessing grittiness. Duckworth and Yeager (2015), for instance, have enumerated teacher-report measures and performance task assessment as potential tools to evaluate non-cognitive abilities in school contexts. Biographical evidence of long-term commitments can also serve as a complementary approach to assess individuals' determination to accomplish challenging longstanding aspirations (Robertson-Kraft and Duckworth, 2014). Other researchers (Bowman et al.,) have recommended the use of personal statements and documents indicating academic and extracurricular engagements of individuals to assess individuals' grittiness.

Moreover, it is also essential to continuously examine the validity of grit measures based on alternative statistical approaches, such as an extended procedure of CFA conducted in the investigation of Schmidt et al. (2018) and IRT analyses. To date, there were few studies that adopted IRT-based approaches to investigate the psychometric properties of existing grit scales (Areepattamannil and Khine, 2018; Tyumeneva et al., 2019; Gonzalez et al., 2020). Further, these studies primarily concentrated on providing psychometric information about Grit-O and Grit-S, so findings have limited implications for evaluating the validity of other grit scales, such as TMGS (Datu et al., 2017a) and Academic Grit Scale (Clark and Malecki, 2019). Adopting more sophisticated analytic procedures (e.g., polytomous IRT modeling techniques and extended versions of CFA) can generate stronger evidence on the psychometric validity of existing grit measures.

### Strengthening Evidence on the Incremental Validity of Grit

Given the empirical evidences dampening the incremental validity of grit above and beyond the effects of theoretically related constructs, such as conscientiousness (Maddi et al., 2013; Ivcevic and Brackett, 2014; Rimfeld et al., 2016; Credé et al., 2017; Muenks et al., 2017) and academic variables, such as motivation and engagement (Steinmayr et al., 2018), there is a need to conduct additional studies on how refined models of grit can uniquely contribute to desirable performance outcomes beyond and above the effects of theoretically relevant constructs (i.e., Big Five personality factors, self-control, self-efficacy, and motivation). In addition, it is equally important to the unique predictive power of grit on psychological and physical health after controlling for pertinent covariates (e.g., neuroticism, self-discipline, and eating behaviors).

Furthermore, although other studies have shown that the original model of grit can predict retention beyond the influence of traditional variables that relate to admission in college programs (e.g., high school GPA and college GPA) in selected undergraduate students in the United States (Saunders-Scott et al., 2018) and cadets in West Point (Duckworth et al., 2007), not so much is known on how alternative models of grit predict retention outcomes in non-WEIRD societies. It is therefore essential to carry out investigations on the incremental validity of triarchic model of grit (Datu et al., 2017a, 2018a) and revised model of grit (Jachimowicz et al., 2018) on retention outcomes in different domains of performance (e.g., sports, post-graduate degree, extracurricular activities, and artistic activities).

Future research can also generate stronger evidence on the construct validity of grit through exploring how grit and its dimensions may relate to specific neurocognitive and physiological processes. For instance, research shows that perseverance of effort is associated with smaller mean difference in N1 amplitude for double cue trials, which suggest higher levels of sustained attention (Kalia et al., 2018) and neural activities in the medial prefrontal cortex (Myers et al., 2016). Moreover, future research can explore the connection of grit to objective indicators of optimal physical health, such as frequency of visits to physicians, regulation of antibody genes, and actual blood pressure. Studies of these kinds can provide more rigorous and convincing insights about the predictive power of grit in various domains of performance and well-being.

### Adopting Alternative Methodological Approaches

Except for a few investigations that used longitudinal (O'Neal, 2018; O'Neal et al., 2018; Park et al., 2018; Jiang et al., 2019; Datu et al., 2020) and experimental (Lucas et al., 2015) research approaches, previous studies mainly relied on cross-sectional research designs in examining the role of grit in performance, optimal psychological, and well-being outcomes. Given that cross-sectional designs are prone to common method bias that can delimit the validity of such studies (Podsakoff et al., 2003), future investigations are encouraged to adopt longitudinal designs (e.g., cross-lagged panel and latent growth curve modeling approaches) to offer stronger evidence about the complex association of grit with desirable performance and well-being. Furthermore, the extant grit literature may profit from conducting person-oriented approaches to explore how various profiles of grit may relate to optimal levels of achievement in different domains of performance. The use of experimental research design is needed to provide cause evidence on the effects of grit to achievement and well-being outcomes.

As majority of published studies on grit examined the benefits of grit in student populations, future research can also explore how grit may foster meaningful outcomes in clinical populations. Past investigations have already initiated exciting avenues for unpackaging the appealing values of grittiness in specialized populations, such as college students with ADHD (Gray et al., 2015) and patients with substance dependence (Griffin et al., 2016). Consistent with this point of contention, previous research (Griffin et al., 2016) points out that integrating grit in motivating clients to achieve optimal recovery can complement existing clinical psychological interventions. Hence, it is a promising research initiative to investigate the effects of grit on the lives of clients with diverse medical conditions.

### Identifying Antecedents of Grit

There is a need to explore how specific social and contextual factors relate to grit. Recognizing the importance of interpersonal factors on passion and perseverance for long-term goals, research demonstrates that relationship with peers (Lan and Moscardino, 2019) and school connectedness (Renshaw and Bolognino, 2016) are linked to higher levels of grit. Even classroom-level variables, such as classroom peer grit (O'Neal, 2018) and perceived mastery–approach and the capacity of classroom to promote mastery–approach goals (Park et al., 2018) are linked to increased grit in student populations. In fact, classroom peer grit has stronger influence than individual-level grit on subsequent literacy achievement even after controlling for previous literacy achievement and relevant demographic covariates, such as age, gender, and home language (O'Neal, 2018). However, in the context of Latino undergraduate students, perceived family support did not contribute to grit (Vela et al., 2015). Moreover, there is no evidence yet that report how specific academic and non-academic policies or programs in school contribute to development of grit in academic settings. Indeed, more studies are desired to explore how different social factors catalyze grit, as well as precise psychological mechanisms linking, such as social, contextual, and interpersonal variables to grit construct.

### Exploring the “Dark Side” of Grit

Existing investigations indirectly point to the caveats of embodying a sustained interest and perseverance to achieve goals even in the face of failures. Consistent with this perspective, a series of experiments carried out by Lucas et al. (2015) has shown that grittier individuals exerted more effort than their less gritty counterparts when finding solutions to unsolvable tasks at the expense of accomplishing fewer duties (Study 1) and when losing a game (Study 2). Moreover, when grittier participants were given an opportunity to quit, they showed greater persistence and stayed in a losing game. Indeed, these results indicate that there are potentially damaging psychological costs associated with espousing grit especially in contexts where failure is bound to happen. Similarly, espousing higher grit and tendency to give up on difficult or boring activities (i.e., perseverance dimension of impulsivity) has been linked to more incidence of suicidal attempts (Anestis and Selby, 2015). This evidence points to the potential maladaptive effects of grit on well-being outcomes. Future investigations, therefore, are needed to explore the adverse “side effects” of grittiness and specific contextual conditions that may amplify the “dark side” of grit in various domains of performance and psychological functioning.

### Addressing Cultural Biases in Grit

Many issues revolving around the “cultural biasness” of grit remain unresolved. One of the most controversial problems points to grit as a “racist” construct that characterized motivational intensity for distal goals for individuals belonging to families with high socioeconomic status (Herold, 2015). In a similar vein, McGee and Stovall (2015) have criticized extant grit theory for being a “racist” construct that fails to capture unique mental health needs of Black students in predominantly White educational institutions. Future researchers can eventually address these conceptual speculations on the cultural insensitivity of grit construct through exploring the invariance of specific grit models across individuals from diverse economic and racial backgrounds, as well as examining the moderating role of socioeconomic status and racial backgrounds on the hypothesized link of grit to positive performance and well-being outcomes in different settings. Because the present grit literature is dominated by studies carried out in WEIRD societies, more investigations are necessary to explore how grit operates in non-WEIRD contexts, which, in turn, generate solid evidence regarding the cross-cultural generalizability of grit.

## Theoretical Implications

Before discussing the anticipated theoretical contributions of this review, it is important to note its limitations. Given that studies included in this review were not done in a systematic manner, it is likely that this review might be prone to selection bias. Future reviews can address this methodological shortcoming through conducting systematic reviews that involve organized and careful selection of studies based on a specific range of inclusion criteria. In addition, the qualitative nature of this review precludes concrete insights on the magnitude of relationship between grit and well-being or other positive health outcomes. Future researchers can fill this gap through carrying out meta-analyses in order to quantitatively summarize the effects sizes between grit and a wide range of optimal outcomes. In order to generate more robust and comprehensive evidence regarding the mental health, physical, and neural correlates of grit, future research may integrate the methodological strengths of qualitative (e.g., systematic) and quantitative (e.g., meta-analyses) reviews through performing a systematic and meta-analytic review. Further, given the limited evidence about the incremental validity of alternative models of grit (e.g., triarchic model of grit) above and beyond the effects of relevant psychological constructs, such as conscientiousness and self-control, caution should be observed when interpreting conclusions about the educational and psychological payoffs associated with these frameworks of grit.

This integrative review has implications for advancing research programs about the generalizability, measurement, antecedents, and consequences of grit. First, although prior reviews (Credé et al., 2017; Datu et al., 2017a,b; Credé, 2018; Lam and Zhou, 2019) primarily concentrated on summarizing the link of grit to academic performance and relevant outcomes, this review offers a more holistic overview about the correlates of grit through providing substantive summary on how grit and its dimensions predict optimal psychological, mental health, and physiological outcomes. Drawing from previous scientific findings about the consequences and correlates of grit, this review also organizes cognitive, affective, behavioral, and social mechanisms underscoring the educational, organizational, and mental health benefits of grit into the optimal performance and health (OPAH) model of grit. Second, unlike previous reviews on grit that offered broad insights on how to address conceptual and measurement issues on grit, this review outlines a few specific recommendations on how to address existing conceptual and psychometric issues in the grit construct. Future researchers may consider such suggestions in order to design more culturally nuanced grit scales that can capture individual difference in persistence toward accomplishing long-term ambitions in various cultural contexts. Third, while past review articles focused on summarizing studies about the benefits of grit (Datu et al., 2017b; Lam and Zhou, 2019), these reviews provide scant insights about the importance of exploring maladaptive impacts of staying gritty. This review addresses this limitation through briefly rationalizing the significance of pinpointing the disadvantages associated with grittiness as a future scholarly initiative.

## Practical Implications

This review carries valuable implications for educational and mental health practitioners in various contexts. Given the mixed evidence regarding the psychometric validity and construct validity of the Grit-S and Grit-O (Muenks et al., 2017; Areepattamannil and Khine, 2018; Tyumeneva et al., 2019), educational policy makers and administrators (e.g., principals, vice principals, admission office director, and subject area coordinators) should practice caution when integrating these questionnaires as assessment tools in high-stake academic activities and career placements. Further, as most studies appear to indicate that only perseverance of effort serves as a consistent predictor of many key educational outcomes, such as academic achievement (Chang, 2014; Wolters and Hussain, 2015; Muenks et al., 2017; Steinmayr et al., 2018), motivation (Eskreis-Winkler et al., 2014; Datu et al., 2018b), engagement (Datu et al., 2016), and achievement goal orientation (Chen et al., 2018), schools are encouraged to invest in psychological programs that aim to cultivate persistence among typically developing and at-risk students. To date, there have been a number of educational interventions (see Alan et al., 2020) that provided promising evidence about the effects of these initiatives on key learning outcomes.

School-based and community mental health practitioners are also likely to benefit from the findings of this review. As prior studies have demonstrated the mental health benefits of grit's dimensions such as perseverance (Pierro et al., 2011; Lee, 2017; Hwang et al., 2018) and adaptability (Datu et al., 2018b, 2020; Datu and Restubog, 2020; Datu and Zhang, 2020), school psychologists and guidance counselors are recommended to consider designing school-wide psychoeducational interventions that aim to promote these personal qualities in student populations. The extant literature on the association of grit with optimal neural activities (Myers et al., 2016; Kalia et al., 2018; Wang et al., 2018) points to the potential benefits of initiating interdisciplinary collaborations between neuroscientists and educational practitioners (e.g., teachers and subject area coordinators) in order to build grit interventions that can optimize effective learning and psychological health.

## Conclusions

There is an ongoing debate on the theoretical validity and benefits of fostering grit in various contexts. This integrative review contributes to extant grit literature through (a) discussing concrete issues on the measurement, validity, and correlates of grit; (b) reviewing alternative models of grit; and (c) discussing how these alternative models of grit can address existing issues on the generalizability and measurement of grit. Much like other valuable psychological constructs, better theorizing, measurement, and methodological approaches can address skepticisms raised against grit. Indeed, understanding the complex nature of grit goes beyond investigating the roles of passion and perseverance in pursuing long-term goals.

## Author Contributions

The author confirms being the sole contributor of this work and has approved it for publication.

## Conflict of Interest

The author declares that the research was conducted in the absence of any commercial or financial relationships that could be construed as a potential conflict of interest.

## References

[B1] Abuhassàn A. Bates T. C. (2015). Grit: distinguishing effortful persistence from conscientiousness. J. Individ. Differ. 36, 205–214. 10.1027/1614-0001/a000175

[B2] Akos P. Kretchmar J. (2017). Investigating grit at a non-cognitive predictor of college success. Rev. High. Educ. 40, 163–186. 10.1353/rhe.2017.0000

[B3] Alan S. Boneva T. Ertac S. (2020). Ever failed, try again, succeed better: results from a randomized educational intervention on grit. Q. J. Econ. 134, 1211–1262. 10.1093/qje/qjz006

[B4] Anestis M. D. Selby E. A. (2015). Grit and perseverance in suicidal behavior and non-suicidal self-injury. Death Studies 39, 211–218. 10.1080/07481187.2014.946629 25611458

[B5] Antonovsky H. Sagy S. (1986). The development of a sense of coherence and its impact on responses to stress situations. J. Soc. Psychol. 126, 213–225. 3724090

[B6] Aparicio M. Bacao F. Oliveira T. (2017). Grit in the path to e-learning success. Comput. Hum. Behav. 66, 388–389. 10.1016/j.chb.2016.10.009

[B7] Areepattamannil S. Khine M. S. (2018). Evaluating the psychometric properties of the original grit scale using rasch analysis in an Arab adolescent sample. J. Psychoeduc. Assess. 36, 856–862. 10.1177/0734282917719976

[B8] Armstrong A. van der Lingen E. Lourens R. Chen J. Y. J. (2018). Towards a new model of grit within a cognitive-affective framework of self-regulation. S. Afr. J. Bus. Manage. 49:8 10.4102/sajbm.v49i1.13

[B9] Baumeister R. F. Campbell J. D. Krueger J. I. Vohs K. D. (2003). Does high self-esteem cause better performance, interpersonal success, happiness, or healthier lifestyles? Psychol. Sci. Public Interest 4, 1–44. 10.1111/1529-1006.01431 26151640

[B10] Blalock D. V. Young K. C. Kleiman E. M. (2015). Stability amidst turmoil: grit buffers the effects of negative life events on suicidal ideation. Psychiatry Res. 228, 781–784. 10.1016/j.psychres.2015.04.041 26070767

[B11] Bowman N. A. Hill P. L. Denson N. Bronkema R. (2015). Keep on truckin' or stay the course? Exploring grit dimensions as differential predictors of educational achievement, satisfaction, and intentions. Soc. Psychol. Pers. Sci. 6, 639–645. 10.1177/1948550615574300

[B12] Burkhart R. A. Tholey R. M. Guinto D. Yeo C. J. Chojnacki K. A. (2014). Grit: a marker of residents at risk for attrition. Surgery 155, 1014–1022. 10.1016/j.surg.2014.01.015 24856121

[B13] Camp C. L. Wang D. Turner N. S. Grawe B. M. Kogan M. Kelly A. M. (2018). Objective predictors of grit, self-control, and conscientiousness in orthopaedic surgery residency applicants. J. Am. Acad. Orthopaed. Surg. 27, e227–e234. 10.5435/jaaos-d-17-00545 30247313

[B14] Carlson M. Wilcox R. Chou C. P. Chang M. Yang F. Blanchard J.. (2011). Psychometric properties of reverse-scored items on the CES-D in a sample of ethnically diverse older adults. Psychol. Assess. 23, 558–562. 10.1037/a0022484 21319906 PMC3115428

[B15] Caza A. Posner B. Z. (2019). How and when does grit influence leaders' behavior? Leadersh. Organ. Dev. J. 40, 124–134. 10.1108/LODJ-06-2018-0209

[B16] Ceschi A. Sartori R. Dickert S. Costantini A. (2016). Grit or honesty-humility? New insights into the moderating role of personality between the health impairment process and counterproductive work behavior. Front. Psychol. 7:1799. 10.3389/fpsyg.2016.01799 28018250 PMC5147463

[B17] Chang W. (2014). Grit and academic performance: is being grittier better? (Doctoral dissertation). Retrieved from Open Access Dissertations (Paper 1306).

[B18] Chen C. Ye S. Hangen E (2018) Predicting achievement goals in the East West: the role of grit among American Chinese university students. Educ. Psychol. 38, 820–837. 10.1080/01443410.2018.1458975

[B19] Clark K. N. Malecki C. K. (2019). Academic grit scale: psychometric properties and associations with achievement and life satisfaction. J. School Psychol. 72, 49–66. 10.1016/j.jsp.2018.12.001 30819462

[B20] Constantin T. Holman A. Hojbotâ A. M. (2011). Development and validation of a motivational persistence scale. Psihologija 45, 99–120. 10.2298/PSI1202099C

[B21] Cormier D. L. Dunn J. G. H. Dunn J. C. (2019). Examining the domain specificity of grit. Pers. Individ. Differ. 139, 349–354. 10.1016/j.paid.2018.11.026

[B22] Cosgrove J. M. Chen Y. T. Castelli D. M. (2018). Physical fitness, grit, school attendance, and academic performance among adolescents. BioMed Res. Int. 2018:9801258. 10.1155/2018/9801258 29568776 PMC5820665

[B23] Credé M. (2018). What shall we do about grit? A critical review of what we know and what we don't know. Educ. Res. 47, 606–611. 10.3102/0013189X18801322

[B24] Credé M. (2019). Total grit score does not represent perseverance. Proc. Natl. Acad. Sci. U.S.A. 116:3941. 10.1073/pnas.1816934116 30808813 PMC6410808

[B25] Credé M. Tynan M. C. Harms P. D. (2017). Much ado about grit: a meta-analytic synthesis of the grit literature. J. Pers. Soc. Psychol. 113, 492–511. 10.1037/pspp0000102 27845531

[B26] Datu J. A. D. Restubog S. L. D (2020). The emotional pay-off of staying gritty: linking grit with social-emotional learning and affective well-being. Br. J. Guid. Counsel. 48, 697–708. 10.1080/03069885.2020.1758922

[B27] Datu J. A. D. (2017). Sense of relatedness is linked to higher grit in a collectivist setting. Pers. Individ. Differ. 105, 135–138. 10.1016/j.paid.2016.09.039

[B28] Datu J. A. D. Fong R. W. (2018). Examining the association of grit with test emotions among Hong Kong Chinese primary school students. School Psychol. Int. 39, 510–525. 10.1177/0143034318793468

[B29] Datu J. A. D. King R. B. Valdez J. P. M. Eala M. S. (2019). Grit is associated with lower depression via meaning in life among Filipino high school students. Youth Soc. 51, 865–876. 10.1177/0044118X18760402

[B30] Datu J. A. D. McInerney D. M. (2017). Does culture matter for grit? Mapping cross-cultural directions in grit research programs, in Self–Driving Positive Psychology and Well-being: International Advances in Self Research, Vol. 6, eds Guay F. Marsh H. W. Craven R. G. McInerney D. M. (Charlotte, NC: Information Age Publishing), 113–133.

[B31] Datu J. A. D. McInerney D. M. Żemojtel-Piotrowska M. Hitokoto H. Datu N. (2020). Is grittiness next to happiness? Examining the association of triarchic model of grit dimensions with well-being outcomes. J. Happiness Stud. 10.1007/s10902-020-00260-629927277

[B32] Datu J. A. D. Valdez J. P. M. King R. B. (2016). Perseverance counts but consistency does not! Validating the Short-Grit Scale in a collectivist setting. Curr. Psychol. 35, 121–130. 10.1007/s12144-015-9374-2

[B33] Datu J. A. D. Yuen M. Chen G. (2017a). Development and validation of the triarchic model of grit scale (TMGS): evidence from Filipino undergraduate students. Pers. Individ. Differ. 114, 198–205. 10.1016/j.paid.2017.04.012

[B34] Datu J. A. D. Yuen M. Chen G. (2017b). Grit and determination: a review of literature with implications for theory and research. J. Psychol. Counsel. Schools 27, 168–176. 10.1017/jgc.2016.2

[B35] Datu J. A. D. Yuen M. Chen G. (2018a). Exploring determination for long-term goals in a collectivist context: a qualitative study. Curr. Psychol. 37, 263–271. 10.1007/s12144-016-9509-0

[B36] Datu J. A. D. Yuen M. Chen G. (2018b). The triarchic model of grit is linked to academic success and well-being among Filipino high school students. School Psychol. Q. 33, 428–438. 10.1037/spq0000234 29927277

[B37] Datu J. A. D. Zhang J. (2020). Validating the Chinese version of triarchic model of grit scale in technical-vocational college students. J. Psychoeduc. Assess. 10.1177/0734282920974813. [Epub ahead of print].

[B38] Deci E. L. Ryan R. M. (2000). The “what” and “why” of goal pursuits: human needs and the self-determination of behavior. Psychol. Inq. 11, 227–268. 10.1207/S15327965PLI1104_01

[B39] DiMenichi B. C. Richmond L. L. (2015). Reflecting on past failures leads to increased perseverance and sustained attention. J. Cogn. Psychol. 27, 180–193. 10.1080/20445911.2014.995104

[B40] Disabato D. J. Goodman F. R. Kashdan T. B. (2019). Is grit relevant to well-being and strengths? Evidence across the globe for separating perseverance of effort and consistency of interests. J. Pers. 87, 194–211. 10.1111/jopy.12382 29573415

[B41] Disabato D. J. Goodman F. R. Kashdan T. B. Short J. L. Jarden A. (2016). Different types of well-being? A cross-cultural examination of hedonic and eudaimonic well-being. Psychol. Assess. 28, 471–482. 10.1037/pas0000209 26348031

[B42] Dreisbach G. Fröber K. (2019). On how to be flexible (or not): modulation of the stability-flexibility balance. Curr. Direct. Psychol. Sci. 28, 3–9. 10.1177/0963721418800030

[B43] Duckworth A. Gross J. J. (2014). Self-control and grit related but separable determinants of success. Curr. Direct. Psychol. Sci. 23, 319–325. 10.1177/0963721414541462 26855479 PMC4737958

[B44] Duckworth A. L. Eskreis-Winkler L. (2015). Grit, in International Encyclopedia of the Social & Behavioral Sciences, 2nd Edn., Vol. 10, ed Wright J. D. (Oxford: Elsevier), 397–401. 10.1016/B978-0-08-097086-8.26087-X

[B45] Duckworth A. L. Kirby T. Tsukayama E. Berstein H. Ericsson K. A. (2010). Deliberate practice spells success: why grittier competitors triumph at the National Spelling Bee. Soc. Psychol. Pers. Sci. 2, 174–181. 10.1177/1948550610385872

[B46] Duckworth A. L. Peterson C. Matthews M. D. Kelly D. R. (2007). Grit: perseverance and passion for long-term goals. J. Pers. Soc. Psychol. 92, 1087–1101. 10.1037/0022-3514.92.6.1087 17547490

[B47] Duckworth A. L. Quinn P. D. (2009). Development and validation of the Short Grit Scale (Grit-S). J. Pers. Assess. 91, 166–174. 10.1080/00223890802634290 19205937

[B48] Duckworth A. L. Yeager D. S. (2015). Measurement matters: assessing personal qualities other than cognitive ability for educational purposes. Educ. Res. 44, 237–251. 10.3102/0013189X15584327 27134288 PMC4849415

[B49] Dumfart B. Neubauer A. C. (2016). Conscientiousness is the most powerful noncognitive predictor of school achievement in adolescents. J. Individ. Differ. 37, 8–15. 10.1027/1614-0001/a000182

[B50] Ebadi S. Weisi H. Khaksar Z. (2018). Developing an Iranian ELT context-specific grit instrument. J. Psycholing. Res. 47, 975–997. 10.1007/s10936-018-9571-x 29511915

[B51] Erikson E. H. (1982). The Life Cycle Completed. New York, NY: W. W. Norton & Company, Inc.

[B52] Eskreis-Winkler L. Gross J. J. Duckworth A. L. (2016). Grit: sustained self-regulation in the service of superordinate goals, in Handbook of Self-Regulation: Research, Theory and Applications. 16, eds Vohs K. D. Baumeister R. F. (New York, NY: Guilford), 380–395.

[B53] Eskreis-Winkler L. Shulman E. P. Beal S. A. Duckworth A. L. (2014). The grit effect: predicting retention in the military, the workplace, school and marriage. Front. Psychol. 5:36. 10.3389/fpsyg.2014.00036 24550863 PMC3910317

[B54] Feldman J. L. Freitas A. L. (2016). An investigation of the reliability and self-regulatory correlates of conflict adaptation. Exp. Psychol. 63, 237–247. 10.1027/1618-3169/a000328 27750519

[B55] Fernández F. D. Arco J. L. Hervás M. (2020). Grit as a predictor and outcome of educational, professional, and personal success: a systematic review. Psicol. Educ. 26, 163–173. 10.5093/psed2020a11

[B56] Gilchrist J. D. Fong A. J. Herbison J. D. Sabiston C. M. (2018). Feelings of pride are associated with grit in student-athletes and recreational runners. Psychol. Sport Exerc. 36, 1–7. 10.1016/j.psychsport.2017.12.009

[B57] Gonzalez O. Canning J. R. Smyth H. MacKinnon D. P. (2020). A psychometric evaluation of the Short Grit Scale: a closer look at its factor structure and scale functioning. Eur. J. Psychol. Assess. 36, 646–657. 10.1027/1015-5759/a000535 PMC8034360 33840984

[B58] Gray S. A. Fettes P. Woltering S. Mawjee K. Tannock R. (2015). Symptom manifestation and impairments in college students with ADHD. J. Learn. Disabil. 49, 616–630. 10.1177/0022219415576523 25778457

[B59] Griffin M. L. McDermott K. A. McHugh R. K. Fitzmaurice G. M. Weiss R. D. (2016). Grit in patients with substance use disorders. Am. J. Addict. 25, 652–658. 10.1111/ajad.12460 27759947 PMC5484735

[B60] Gross J. J. Levenson R. W. (1997). Hiding feelings: the acute effects of inhibiting positive and negative emotions. J. Abnorm. Psychol. 106, 95–103. 10.1037//0021-843x.106.1.95 9103721

[B61] Guerrero L. R. Dudovitz R. Chung P. J. Dosanjh K. K. Wong M. D. (2016). Grit: a potential protective factor against the use and other risk behaviors among Latino adolescents. Adolesc. Subst. Abuse 16, 275–281. 10.1016/j.acap.2015.12.016 26796576 PMC4821776

[B62] Hagger M. S. Hamilton K. (2019). Grit and self-discipline as predictors of effort and academic attainment. Br. J. Educ. Psychol. 89, 324–342. 10.1111/bjep.12241 30101970

[B63] Hammer J. H. Good G. E. (2010). Positive psychology: an empirical examination of beneficial aspects of endorsement of masculine norms. Psychol. Men Mascul. 11, 303–318. 10.1037/a0019056

[B64] Henrich J. Heine S. J. Norenzayan A. (2010). Most people are not WEIRD. Nature 466:29. 10.1038/466029a 20595995

[B65] Herold B. (2015). Is ‘Grit’ Racist? Digital Education. Retrieved from: http://blogs.edweek.org/edweek/DigitalEducation/2015/01/is_grit_racist.html

[B66] Hill P. L. Burrow A. L. Bronk K. C. (2014). Persevering with positivity and purpose: an examination of purpose commitment and positive affect as predictors of grit. J. Happ. Stud. 17, 257–269. 10.1007/s10902-014-9593-5

[B67] Hodge B. Wright B. Bennett P. (2018). The role of grit in determining engagement and academic outcomes for university students. Res. High. Educ. 59, 448–460. 10.1007/s11162-017-9474-y

[B68] Hodge D. R. Hong P. Y. P. Choi S. (2019). Spirituality, employment hope, and grit: modeling the relationship among underemployed urban African Americans. Soc. Work Res. 43, 43–52. 10.1093/swr/svy034

[B69] Howard J. M. Nicholson B. C. Chesnut S. R. (2019). Relationships between positive parenting, overparenting, grit, and academic success. J. Coll. Stud. Dev. 60, 189–202. 10.1353/csd.2019.0018

[B70] Hwang M. H. Lim H. J. Ha H. S. (2018). Effects of grit on the academic success of adult female students at Korean Open University. Psychol. Rep. 121, 705–725. 10.1177/0033294117734834 29298549

[B71] Ion A. Mindu A. Gorbănescu A. (2017). Grit in the workplace: hype or ripe? Pers. Individ. Differ. 111, 163–168. 10.1016/j.paid.2017.02.012

[B72] Ivcevic Z. Brackett M. (2014). Predicting school success: comparing conscientiousness, grit, and emotion regulation ability. J. Res. Pers. 52, 29–36. 10.1016/j.jrp.2014.06.005

[B73] Jachimowicz J. M. Wihler A. Bailey E. R. Galinksy A. D. (2018). Why grit requires perseverance and passion to positively predict performance. Proc. Natl. Acad. Sci. U.S.A. 40, 9980–9985. 10.1073/pnas.1803561115 30224491 PMC6176608

[B74] Jiang W. Xiao Z. Liu Y. Guo K. Jiang J. Du X. (2019). Reciprocal relations between grit and academic achievement: a longitudinal study. Learn. Individ. Differ. 71, 13–22. 10.1016/j.lindif.2019.02.004

[B75] Jin B. Kim J. (2017). Grit, basic needs satisfaction, and subjective well-being. J. Individ. Differ. 38, 29–35. 10.1027/1614-0001/a000219

[B76] Jordan M. H. Teasley T. J. G. Walker W. J. Schraeded M. (2015). An integrative approach to identifying factors related to long-term career commitments. Career Dev. Int. 20, 163–178. 10.1108/CDI-05-2013-0071

[B77] Kalia V. Thomas R. Osowski K. Drew A. (2018) Staying alert? Neural correlates of the association between grit and attention networks. Front. Psychol. 9:1377. 10.3389/fpsyg.2018.01377 30123173 PMC6085581

[B78] Kannangara C. S. Allen W. R. E. Nahar N. Khan S. Z. N. Rogerson S. Carson J. (2018). All that glitters is not grit: three studies of grit in university students. Front. Psychol. 9:1539. 10.3389/fpsyg.2018.01539 30210389 PMC6123604

[B79] Kashdan T. B. Rottenberg J. (2010). Psychological flexibility as a fundamental aspect of health. Clin. Psychol. Rev. 30, 865–878. 10.1016/j.cpr.2010.03.001 21151705 PMC2998793

[B80] Kern M. L. Benson L. Steinberg E. A. Steinberg L. (2016). The EPOCH measure of adolescent well-being. Psychol. Assess. 28, 586–597. 10.1037/pas0000201 26302102

[B81] Kleiman E. M. Adams L. M. Kashdan T. B. Riskind J. H. (2013). Gratitude and grit indirectly reduce risk of suicidal ideations by enhancing meaning in life: evidence for a mediated moderation model. J. Res. Pers. 47, 539–546. 10.1016/j.jrp.2013.04.007

[B82] Knauft K. Ortiz S. Velkoff E. Smith A. Kalia A. (2019). Keep calm and carry on? Grit buffers against disordered eating unless expressive suppression is used to regulate emotions. J. Soc. Clin. Psychol. 38, 321–342. 10.1521/jscp.2019.38.4.321

[B83] Lam K. L. L. Zhou M. (2019). Examining the relationship between grit and academic achievement within K-12 and higher education: a systematic review. Psychol. Schools 56, 1654–1686. 10.1002/pits.22302

[B84] Lan X. Moscardino U. (2019). Direct and interactive effects of perceived teacher-student relationship and grit on student wellbeing among stay-behind early adolescents in urban China. Learn. Individ. Differ. 69, 129–137. 10.1016/j.lindif.2018.12.003

[B85] Larkin P. O'Connor D. Williams A. (2015). Does grit influence sport-specific engagement and perceptual-cognitive expertise in elite youth soccer? J. Appl. Sport Psychol. 28, 129–138. 10.1080/10413200.2015.1085922

[B86] Lee T. H. Duckworth A. L. (2018). Organizational grit. Harv. Bus. Rev. 96, 98–105.

[B87] Lee W. W. S. (2017). Relationships among grit, academic performance, perceived academic failure, and stress in associate degree students. J. Adolesc. 60, 148–152. 10.1016/j.adolescence.2017.08.006 28886409

[B88] Li J. Fang M. Wang W. Sun G. Cheng Z. (2018a). The influence of grit on life satisfaction: self-esteem as a mediator. Psychol. Belg. 58, 51–66. 10.5334/pb.400 30479807 PMC6194520

[B89] Li J. Lin L. Zhao Y. Chen J. Wang S. (2018b). Grittier Chinese adolescents are happier: the mediating role of mindfulness. Pers. Individ. Differ. 131, 232–237. 10.1016/j.paid.2018.05.007

[B90] Li J. Zhao Y. Kong F. Du S. Yang S. Wang S. (2018c). Psychometric assessment of the short grit scale among Chinese adolescents. J. Psychoeduc. Assess. 36, 291–296. 10.1177/0734282916674858

[B91] Li J. Zhao Y. Lin L. Chen J. Wang S. (2018d). The freedom to persist: Belief in free will predicts perseverance for long-term goals among Chinese adolescents. Pers. Individ. Differ. 121, 7–10. 10.1016/j.paid.2017.09.011

[B92] Lovering M. E. Heaton K. J. Banderet L. E. Neises K. Andrews J. Cohen B. S. (2015). Psychological and physical characteristics of U. S. marine recruits. Mil. Psychol. 27, 261–275. 10.1037/mil0000082

[B93] Lucas G. M. Gratch J. Cheng L. Marsella S. (2015). When the going gets tough: grit predicts costly perseverance. J. Res. Pers. 59, 15–22. 10.1016/j.jrp.2015.08.004

[B94] Maddi S. R. Erwin L. M. Carmody C. L. Villareal B. J. White M. Gundersen K. K. (2013). Relationship of hardiness, grit, and emotional intelligence to internet addiction, excessive consumer spending, and gambling. J. Posit. Psychol. 8, 128–134. 10.1080/17439760.2012.758306

[B95] Martin J. J. Byrd B. Watts M. L. Dent M. (2015). Gritty, hardy, and resilient: predictors of sport engagement and life satisfaction. J. Clin. Sport Psychol. 9, 345–359. 10.1123/jcsp.2015-0015

[B96] Mayer J. D. Skimmyhorn W. (2017). Personality attributes that predict cadet performance at West Point. J. Res. Pers. 66, 14–26. 10.1016/j.jrp.2016.10.012

[B97] McClelland D. C. (1961). The Achieving Society. Oxford: Van Nostrand.

[B98] McGee E. O. Stovall D. (2015). Reimagining critical race theory in education: mental health, healing, and the pathway to liberatory praxis. Educ. Theory 65, 491–511. 10.1111/edth.12129

[B99] Meriac J. P. Slifka J. S. LaBat L. R. (2015). Work ethic and grit: an examination of empirical redundancy. Pers. Individ. Differ. 86, 401–405. 10.1016/j.paid.2015.07.009

[B100] Mooradian T. Matzler K. Uzelac B. Bauer F. (2016). Perspiration and inspiration: grit and innovativeness as antecedents of entrepreneurial success. J. Econ. Psychol. 56, 232–243. 10.1016/j.joep.2016.08.001

[B101] Moore R. C. Hussain M. A. Watson C. W. M. Fazeli P. L. Marquine M. J. Yarns B. C.. (2018). Grit and ambition are associated with better neurocognitive and everyday functioning among adults living with HIV. AIDS Behav. 22, 3214–3225. 10.1007/s10461-018-2061-1 29455265 PMC6097949

[B102] Moshier S. J. Szuhany K. L. Hearon B. A. Smits J. A. J. Otto M. W. (2016). Anxiety sensitivity uniquely predicts exercise behaviors in young adults seeking to increase physical activity. Behav. Modif. 40, 178–198. 10.1177/0145445515603704 26342011

[B103] Mueller B. A. Wolfe M. T. Syed I. (2017). Passion and grit: an exploration of the pathways leading to venture success. J. Bus. Ventur. 32, 260–279. 10.1016/j.jbusvent.2017.02.001

[B104] Muenks K. Wigfield A. Yang J. S. O'Neal C. R. (2017). How true is grit? Assessing its relations to high school and college students' personality characteristics, self-regulation, engagement, and achievement. J. Educ. Psychol. 109, 599–620. 10.1037/edu0000153

[B105] Mullen P. R. Crowe A. (2018). A psychometric investigation of the short grit scale with a sample of school counselors. Meas. Eval. Counsel. Dev. 51, 151–162. 10.1080/07481756.2018.1435194

[B106] Musumari P. M Tangmunkongvorakul A. Srithanaviboonchai K. Techasrivichien T. Suguimoto S. P. Ono-Kihara M. (2018) Grit is associated with lower level of depression anxiety among university students in Chiang Mai, Thailand: a cross-sectional study. PLoS ONE 13:e0209121. 10.1371/journal.pone.0209121. 30550597 PMC6294431

[B107] Myers C. A. Wang C. Black J. M. Bugescu N. Hoeft F. (2016). The matter of motivation: striatal resting-state connectivity is dissociable between grit and growth mindset. Soc. Cogn. Affect. Neurosci. 11, 1521–1527. 10.1093/scan/nsw065 27217105 PMC5040906

[B108] Nikolaus C. J. Schierer M. Ellison B. Eicher-Miller H. A. Gundersen C. Nickols-Richardson S. M. (2019). Grit is associated with food security among US parents and adolescents. Am. J. Health Behav. 43, 207–218. 10.5993/AJHB.43.1.17 30522578

[B109] O'Neal C. R. (2018). Individual versus peer grit: influence on later individual literacy achievement of dual language learners. School Psychol. Q. 33, 112–119. 10.1037/spq0000212 28627900

[B110] O'Neal C. R. Goldthrite A. Riley L. W. Atapattu R. K. (2018). A reciprocal, moderated mediation model of grit, engagement, and literacy achievement among dual language learners. Soc. Dev. 27, 665–680. 10.1111/sode.12288

[B111] Park D. Yu A. Baelen R. N. Tsukayama E. Duckworth A. L. (2018). Fostering grit: perceived goal-structure predicts growth in grit and grades. Contemp. Educ. Psychol. 55, 120–128. 10.1016/j.cedpsych.2018.09.007 32831457 PMC7441845

[B112] Pennings S. M. Law K. C. Green B. A. Anestis M. D. (2015). The impact of grit on the relationship between hopelessness and suicidality. Int. J. Cogn. Ther. 8, 130–142. 10.1521/ijct.2015.8.2.130

[B113] Pierro A. Giacomantonio M. Pica G. Kruglanski A. W. Higgins E. T. (2011). On the psychology of time in action: regulatory mode orientations and procrastination. J. Pers. Soc. Psychol. 101, 1317–1331. 10.1037/a0025943 22103579

[B114] Piña-Watson B. López B. Ojeda L. Rodriguez K. M. (2015). Cultural and cognitive predictors of academic motivation among Mexican American adolescents: caution against discounting the impact of cultural processes. J. Multicult. Counsel. Dev. 43, 109–121. 10.1002/j.2161-1912.2015.00068.x

[B115] Podsakoff P. M. MacKenzie S. B. Lee J. Y. Podsakoff N. P. (2003). Common method biases in behavioral research: a critical review of the literature and recommended remedies. J. Appl. Psychol. 88, 879–903. 10.1037/0021-9010.88.5.879 14516251

[B116] Raphiphatthana B. Jose P. Chobthamkit P. (2019). The association between mindfulness and grit: an east vs. west cross-cultural comparison. Mindfulness 10, 146–158. 10.1007/s12671-018-0961-9

[B117] Raphiphatthana B. Jose P. Salmon K. (2018). Does dispositional mindfulness predict the development of grit? J. Individ. Differ. 39, 76–87. 10.1027/1614-0001/a000252

[B118] Reed J. Pritschet B. Cutton D. M. (2013). Grit, conscientiousness, and the transtheoretical model of change for exercise behavior. J. Health Psychol. 18, 612–619. 10.1177/1359105312451866 22904153

[B119] Renshaw T. L. Bolognino S. J. (2016). The college student subjective wellbeing questionnaire: a brief, multidimensional measure of undergraduate's covitality. J. Happ. Stud. 17, 463–484. 10.1007/s10902-014-9606-4

[B120] Rimfeld K. Kovas Y. Dale P. S. Plomin R. (2016). True grit and genetics: predicting academic achievement from personality. J. Pers. Soc. Psychol. 111, 780–789. 10.1037/pspp0000089 26867111 PMC4981570

[B121] Robbins S. B. Allen J. Casillas A. Peterson C. H. Le H. (2006). Unravelling the differential effects of motivational and skills, social, and self-management measures from traditional predictors of college outcomes. J. Educ. Psychol. 98, 598–616. 10.1037/0022-0663.98.3.598

[B122] Roberts B. W. Chernyshenko O. S. Stark S. Goldberg L. R. (2005). The structure of conscientiousness: an empirical investigation based on seven major personality questionnaires. Pers. Psychol. 58, 103–139. 10.1111/j.1744-6570.2005.00301.x

[B123] Robertson-Kraft C. Duckworth A. L. (2014). True grit: trait-level perseverance and passion for long-term goals predicts effectiveness and retention among novice teachers. Teach. Coll. Rec. 116, 1–27. 25364065 PMC4211426

[B124] Ryan R. (1995). Psychological needs and facilitation of integrative processes. J. Pers. 63, 397–427. 10.1111/j.1467-6494.1995.tb00501.x 7562360

[B125] Ryan R. M. Deci E. L. (2000). Self-determination theory and the facilitation of intrinsic motivation, social development, and well-being. Am. Psychol. 55, 68–78. 10.1037/0003-066X.55.1.68 11392867

[B126] Salles A. Cohen G. L. Mueller C. M. (2014). The relationship between grit and resident well-being. Am. J. Surg. 207, 251–254. 10.1016/j.amjsurg.2013.09.006 24238604

[B127] Samson A. C. Proyer R. T. Ceschi G. Pedrini P. P. Ruch W. (2011). The fear of being laughed at in Switzerland regional differences and the role of positive psychology. Swiss J. Psychol. 70, 53–62. 10.1024/1421-0185/a000039

[B128] Saunders-Scott D. Braley M. B. Stennes-Spidahl N. (2018). Traditional and psychological factors associated with academic success: investigating best predictors of college retention. Motiv. Emot. 42, 459–465. 10.1007/s11031-017-9660-4

[B129] Schmidt F. T. C. Fkeckentein J. Retelsdorf J. Eskreis-Winkler L. Möller J. (2019). Measuring grit: a German validation and a domain-specific approach to grit. Eur. J. Psychol. Assess. 35, 436–437. 10.1027/1015-5759/a000407

[B130] Schmidt F. T. C. Nagy G. Fleckenstein J. Möller J. Retelsdorf J. (2018). Same same, but different? Relations between facets of conscientiousness and grit. Eur. J. Pers. 32, 705–720. 10.1002/per.2171

[B131] Sharkey C. M. Bakula D. M. Baraldi A. N. Perez M. N. Suorsa K. I. Chaney J. M.. (2017). Grit, illness-related distress, and psychosocial outcomes in college students with a chronic medical condition: a path analysis. J. Pediatr. Psychol. 43, 552–560. 10.1093/jpepsy/jsx145 29240936

[B132] Sheldon K. M. Jose P. E. Kashdan T. B. Jarden A. (2015). Personality, effective goal-striving, and enhanced well-being: comparing 10 candidate personality strengths. Pers. Soc. Psychol. Bull. 41, 575–585. 10.1177/0146167215573211 25713170

[B133] Silvia P. J. Eddington K. M. Beaty R. E. Nusbaum E. C. Kwapil T. R. (2013). Gritty people try harder: grit and effort-related cardiac autonomic activity during an active coping challenge. Int. J. Psychophysiol. 88, 200–205. 10.1016/j.ijpsycho.2013.04.007 23603450 PMC6176483

[B134] Soto C. J. Kronauer A. Liang J. K. (2016). Five-factor model of personality, in Encyclopedia of Adulthood and Aging, Vol. 2, ed Whitbourne S. K (Hoboken, NJ: Wiley), 506–510.

[B135] Sriram R. Glanzer P. L. Allen C. C. (2018). What contributes to self-control and grit? The key factors in college students. J. Coll. Stud. Dev. 59, 259–273. 10.1353/csd.2018.0026

[B136] Steinmayr R. Weidinger A. F. Wigfield A. (2018). Does students' grit predict their school achievement above and beyond their personality, motivation, and engagement? Contemp. Educ. Psychol. 53, 106–122. 10.1016/j.cedpsych.2018.02.004

[B137] Strayhorn T. L. (2014). What role does grit play in the academic success of black male collegians at Predominantly White Institutions? J Afr Am St. 18, 1–10. 10.1007/s12111-012-9243-0

[B138] Suh E. M. (2007). Downsides of an overly context-sensitive self: implications from the culture and subjective well-being research. J. Pers. 75, 1321–1343. 10.1111/j.1467-6494.2007.00477.x 17995467

[B139] Suzuki Y. Tamesue D. Asahi K. Ishikawa Y. (2015). Grit and work engagement: a cross-sectional study. PLoS ONE 10:e0137501. 10.1371/journal.pone.0137501 26335001 PMC4559397

[B140] Tang X. Wang M. T. Guo J. Salmela-Aro K. (2019). Building grit: the longitudinal pathways between mindset, commitment, grit, and academic outcomes. J. Youth Adolesc. 48, 850–863. 10.1007/s10964-019-00998-0 30788767

[B141] Tedesqui R. A. B. Young B. W. (2017). Investigating grit variables and their relations with practice and skill groups in developing sport experts. High Ability Stud. 28, 167–180. 10.1080/13598139.2017.1340262

[B142] Tovar-García E. D. (2017). The impact of perseverance and passion for long term goals (GRIT) on educational achievements of migrant children: evidence from Tatarstan, Russia. Psicol. Educ. 23, 19–27. 10.1016/j.pse.2017.02.003

[B143] Tucker-Drob E. M. Briley D. A. Engelhardt L. E. Mann F. D. Harden K. P. (2016). Genetically-mediated associations between measures of childhood character and academic achievement. J. Pers. Soc. Psychol. 111, 790–815. 10.1037/pspp0000098 27337136 PMC5073013

[B144] Tyumeneva Y. Kardanova E. Kuzmina J. (2019). Grit: two related but independent constructs instead of one. Evidence from item response theory. Eur. J. Psychol. Assess. 35, 469–478. 10.1027/1015-5759/a000424

[B145] Usher E. L. Li C. R. Butz A. R. Rojas J. P. (2019). Perseverant grit and self-efficacy: are both essential for children's academic success? J. Educ. Psychol. 111, 877–902. 10.1037/edu0000324

[B146] Vainio M. M. Daukantaite D. (2016). Grit and different aspects of well-being: direct and indirect relationships via sense of coherence and authenticity. J. Happ. Stud. 17, 2119–2147. 10.1007/s10902-015-9688-7

[B147] Valdez J. P. M. Datu J. A. D. (2020). How do grit and gratitude relate to flourishing? The mediating role of emotion regulation, in Multidisciplinary Perspectives on Grit: Contemporary Theories, Assessments, Applications, and Critiques, eds van Zyl L. Olckers C. van Der Vaart L. (Springer).

[B148] Vallerand R. J. Blanchard C. M. Mageau G. A. Koestner R. Ratelle C. Léonard M.. (2003). Les passions de l'âme: On obsessive and harmonious passion. J. Pers. Soc. Psychol. 85, 756–767. 10.1037/0022-3514.85.4.756 14561128

[B149] Vazsonyi A. T. Ksinan A. J. Jiskrova G. K. Mikuška J. Javakhishvili M. Cui G. (2019). To grit or not to grit, that is the question! J. Res. Pers. 78, 215–226. 10.1016/j.jrp.2018.12.006

[B150] Vela J. C. Lu M. T. P. Lenz A. S. Hinojosa K. (2015). Positive psychology and familial factors as predictors of Latina/o students' psychological grit. Hispanic J. Behav. Sci. 37, 287–303. 10.1177/0739986315588917

[B151] Vela J. C. Smith W. D. Whittenberg J. F. Guardiola R. Savage M. (2018). Positive psychology factors as predictors of Latina/o college students' psychological grit. J. Multicult. Counsel. Dev. 46, 2–19. 10.1002/jmcd.12089

[B152] Vignoles V. L. Owe E. Becker M. Smith P. B. Easterbrook M. J. Brown R.. (2016). Beyond the ‘east-west’ dichotomy: global variation in cultural models of selfhood. J. Exp. Psychol. Gen. 145, 966–1000. 10.1037/xge0000175 27359126

[B153] Vlasova R. M. Siddarth P. Krause B. Leaver A. M. Laird K. T. Cyr N. S.. (2018). Resilience and white matter integrity in geriatric depression. Am. J. Geriatr. Psychiatry 26, 874–883. 10.1016/j.jagp.2018.04.004 29803529 PMC6086733

[B154] Von Culin K. R. Tsukayama E. Duckworth A. L. (2014). Unpacking grit: motivational correlates of perseverance and passion for long-term goals. J. Posit. Psychol. 9, 306–312. 10.1080/17439760.2014.898320 31404261 PMC6688745

[B155] Walker A. Hines J. Brecknell J. (2016). Survival of the grittiest? Consultant surgeons are significantly grittier than their junior trainees. J. Surg. Educ. 73, 730–734. 10.1016/j.jsurg.2016.01.012 27025568

[B156] Wang S. Dai J. Li J. Wang X. Chen T. Yang X.. (2018). Neuroanatomical correlates of grit: growth mindset mediates the association between gray matter structure and trait grit in late adolescence. Hum. Brain Mapp. 39, 1688–1699. 10.1002/hbm.23944 29331059 PMC6866491

[B157] Wang S. Zhou M. Chen T. Yang X. Chen G. Wang M.. (2017). Grit and the brain: spontaneous activity of the dorsomedial prefrontal cortex mediates the relationship between the trait grit and academic performance. Soc. Cogn. Affect. Neurosci. 12, 452–460. 10.1093/scan/nsw145 27672175 PMC5390743

[B158] Weisskirch R. S. (2018). Grit, self-esteem, learning strategies and attitudes and estimated and achieved course grades among college students. Curr. Psychol. 37, 21–27. 10.1007/s12144-016-9485-4

[B159] Wenner J. R. Randall B. A. (2016). Predictors of prosocial behavior: differences in middle aged and older adults. Pers. Individ. Differ. 101, 322–326. 10.1016/j.paid.2016.05.367 28163344 PMC5287708

[B160] White E. J. Kraines M. A. Tucker R. P. Wingate L. R. Wells T. T. Grant D. M. (2017). Rumination's effects on suicidal ideation through grit and gratitude: a path analysis study. Psychiatry Res. 251, 97–102. 10.1016/j.psychres.2017.01.086 28199915

[B161] Wolters C. A. Hussain M. (2015). Investigating grit and its relations with college students' self-regulated learning and academic achievement. Metacogn. Learn. 10, 293–311. 10.1007/s11409-014-9128-9

[B162] Wood A. M. Linley P. A. Maltby J. Baliousis M. Joseph S. (2008). The authentic personality: a theoretical and empirical conceptualization and the development of the authenticity scale. J. Counsel. Psychol. 55, 385–399. 10.1037/0022-0167.55.3.385

[B163] Yeager D. S. Henderson M. Paunesku D. Walton G. Spitzer B. D'Mello S.. (2014). Boring but important: a self-transcendent purpose for learning fosters academic self-regulation. J. Pers. Soc. Psychol. 107, 559–580. 10.1037/a0037637 25222648 PMC4643833

[B164] Yoshitsu J. Nishikawa K. (2013). Development of the Japanese Grit Scale. Jpn. J. Res. Emot. 20:12 10.2132/personality.24.167

[B165] Zhang X. Noor R. Savalei V. (2016). Examining the effect of reverse worded items on the factor structure of the Need for Cognition Scale. PLoS ONE 11:e0157795. 10.1371/journal.pone.0157795 27305001 PMC4909292

[B166] Zhong C. Wang M. C. Shou Y. Ren F. Zhang X. Li M.. (2018). Assessing construct validity of the Grit-S in Chinese employees. PLoS ONE 13:e0209319. 10.1371/journal.pone.0209319 30576360 PMC6303021

